# Metabolic Fingerprinting of Urine Reveals Metabolite Changes in Women With Breast Cancer

**DOI:** 10.1002/cam4.72018

**Published:** 2026-06-10

**Authors:** Nur Aimi Aliah Zainurin, Anuradha U. K. H. Bambarandhage, Michelle Moreno Escalona, Dimitra Ivanova, Tim Gate, Helen Tench, Manfred Beckman, Helen Phillips, Mandana Pennick, Luis A. J. Mur

**Affiliations:** ^1^ Department of Life Sciences Aberystwyth University Aberystwyth UK; ^2^ Wrexham Maelor Hospital Betsi Cadwaladr University Health Board Wrexham UK; ^3^ Bronglais General Hospital Hywel Dda University Health Board Aberystwyth UK; ^4^ Glan Clwyd Hospital Betsi Cadwaladr University Health Board Bodelwyddan UK

**Keywords:** benign breast disease, breast cancer, plasma, serum, symptom controls, urine

## Abstract

**Background:**

Breast cancer (BC) is one of the most common and serious cancers affecting women worldwide. Mammography facilitates early detection, but improving diagnostic accuracy remains essential to further reduce mortality. This study explores metabolite patterns in urine and blood to see how they differ between women with BC, those with other breast conditions, and healthy individuals.

**Methods:**

Metabolite profiling used direct infusion high‐resolution mass spectrometry (DI‐MS) on urine samples from breast cancer (BC; *n* = 118), benign breast disease (BBD; *n* = 148), symptomatic control (SC; *n* = 95), and healthy control (HC; *n* = 39) groups. Multivariate statistical analyses were performed using the R‐based MetaboAnalyst v6.0 platform and the MixOmics package to compare urine with plasma and serum metabolomes.

**Results:**

Partial least squares‐discriminant analysis (PLS‐DA) suggested poor discrimination between (1) groups (BC vs. HC, BC vs. BBD and BC vs. SC). However, ANOVA (corrected for false discovery rate) targeted urinary metabolites that significantly (*p* < 0.05) differed in BC compared to other groups which included *m/z* suggested to be lactic acid/lactate, lactose 6‐phosphate, D‐ribose sugar, glutamic acid, histidine, L‐valine and urea. PLS‐DA suggested some discrimination with BC histological types (invasive vs. pre‐invasive) and BC molecular subtypes (Luminal A, Luminal B, HER2‐enriched and triple‐negative). Comparisons between urine, serum and plasma metabolomes using Data Integration Analysis for Biomarker Discovery using Latent Components (DIABLO) suggested only moderate correlation. Based on our observations, a network model is proposed linking down‐regulated glutamate/glutamine, histidine, and urea production and increased pentose‐phosphate pathway and lactic acid levels in the BC urine metabolome.

**Conclusion:**

This study suggests that urine metabolomics may improve our understanding of BC development and have potential diagnostic value. These findings warrant validation in larger patient cohorts.

## Graphical abstract

This study uses metabolomics to compare urine, plasma and serum metabolite profiles across breast cancer, benign breast disease, symptomatic and healthy controls. Significant metabolic differences—especially in amino acids—were found between BC and other groups, with moderate correlations across biofluids, suggesting potential for non‐invasive BC diagnostics.

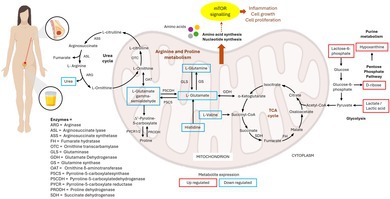

**Abbreviations**
ANOVA: analysis of variance
AU: Aberystwyth University
AUC: area under the curve
BBD: Benign breast disease
BC: breast cancer
BCAA: branched‐chain amino acids
BMI: body mass index
CPMS: Central Portfolio Management System
DAM: differentially accumulating metabolites
DCIS: ductal carcinoma in situ
DHEA: dehydroepiandrosterone sulphate
DIABLO: Data Integration Analysis for Biomarker Discovery using Latent Components
DI‐MS: direct infusion‐mass spectrometry
ER: oestrogen receptor positive
FDR: false discovery rate
GABA: gamma‐aminobutyric acid
GC–qMS: gas chromatography–quadrupole mass spectrometry
H_2_O_2_
: hydrogen peroxide
HC: healthy control
HCRW: Health and Care Research
HPPI‐TOFMS: high‐pressure photon ionisation time‐of‐flight mass spectrometry
HR: Health Research Authority
IBERS: Institute of Biological Environmental and Rural Sciences
IDC: invasive ductal carcinoma
ILC: invasive lobular carcinoma
IMC: invasive mucinous carcinoma
IPC: invasive papillary carcinoma
IRAS: Integrated Research Application System
ITC: invasive tubular carcinoma
KEGG: Kyoto Encyclopaedia of Genes and Genomes
m/z: mass/ion
MSEA: metabolite set enrichment analysis
MSI: mass spectrometry imaging
mTOR: mammalian target of rapamycin
NA: not applicable
NADPH: nicotinamide adenine dinucleotide phosphate
NHS: National Health Services
NMR: nuclear magnetic resonance
PFK1: phosphofructokinase‐1
PLS‐DA: partial least squares‐discriminant analysis
ppm: parts per million
PPP: pentose phosphate pathway
REC: Research Ethics Committee
ROC: receiver operating characteristic curve
ROS: reactive oxygen species
SC: symptomatic control
SD: standard deviation
SMPDB: Small Molecule Pathway Database
TIGAR: TP53 inducible glycolysis and apoptosis regulator
TNBC: triple negative breast cancer
UHPLC‐Q‐TOF‐ESI + MS: ultra‐high performance liquid chromatography‐quadrupole time‐of‐flight‐electrospray ionisation positive mode‐mass spectrometry

## 1 Introduction

Breast cancer (BC) represents a significant global health challenge as the most commonly diagnosed and deadliest cancer among females. Worldwide, the incidence and mortality of BC are projected to increase to 3.6 million and 1.1 million cases, respectively, by 2050. Economically, the global cost of cancers between 2020 and 2050 is projected at $25.2 trillion [1]. In the UK, the National Health Services (NHS) offers a free triennial screening programme for females aged between 50 and 70 years. Screening involves a triple assessment which includes breast examination by the clinician, imaging (mammography, ultrasound and magnetic resonance imaging) as well as biopsy (core needle biopsy, fine needle aspiration and others) [2]. Despite the effectiveness of such screening programmes, limitations include the lower sensitivity of imaging modalities, the risk of misdiagnosis, radiation exposure, discomfort and age [3, 4, 5]. The use of a non‐invasive companion diagnostic could aid decision‐making and triage of urgent cases.

Metabolomics can be considered the nearest 'omic level to phenotype [6] and can provide near real‐time insights into the functional consequences of gene expression. Over the past decades, metabolomics studies have assessed the potential of urine for early BC diagnosis [7]. Among the earliest studies on urinary metabolome for BC diagnosis used gas chromatography–mass spectrometry (GC–MS) to profile the urinary metabolome of women with BC (*n* = 50) and healthy women (*n* = 50). Multivariate classification models identified five potential urinary biomarkers based on the GC–MS spectral peaks. A single feature (191.2261 *m/z* with 535.3876 retention time) which could not be identified showed a highly significant difference between BC and healthy cohorts (*p*‐value = 2.866 × 10^−6^) [7]. With technological developments, high‐pressure photon ionisation time‐of‐flight mass spectrometry (HPPI‐TOFMS) identified acrolein, 2‐pentanone and methyl allyl sulphide as promising biomarkers with sensitivity and specificity of 92.6% and 91.7% in distinguishing between BC (*n* = 24) and healthy controls (*n* = 27), respectively [8]. Similarly, urine samples from BC patients (*n* = 14) and healthy controls (*n* = 16) analysed by liquid chromatography‐mass spectrometry (LC–MS) targeted increases in N‐(2‐furoyl) glycine, histidine and D‐tagatose which were significantly higher (area under the receiver operating characteristic (ROC) curve—area under the curve [AUC] > 0.7) and trigonellinamide, L‐galacto‐2‐heptulose, creatinine and xanthine were lower (AUC ≥ 0.8) in the BC patients compared to healthy controls [9]. Ultra‐high performance liquid chromatography‐quadrupole time‐of‐flight‐electrospray ionisation positive mode‐mass spectrometry (UHPLC‐Q‐TOF‐ESI^+^MS) identified putative biomarkers related to specific BC types, particularly invasive ductal carcinoma (IDC) with oestrogen receptor positive (ER+) from urine and blood samples [10]. A combination of nuclear magnetic resonance (NMR) and gas chromatography–quadrupole mass spectrometry (GC–qMS) platforms was used to profile urine and tissue metabolites from BC (urine, *n* = 30 and tissue, *n* = 30) and cancer‐free (urine of healthy volunteers, *n* = 40 and tissue obtained from the outside margin of BC tissue, *n* = 30). The urinary metabolites α‐hydroxyisobutyrate, glutamine, betaine and hypoxanthine, and the tissue metabolites, lactate, glutamate, valine and taurine, could differentiate between BC urine and tissue from cancer‐free urine and tissue. Interestingly, the urinary metabolites had higher ROC with the AUC of 0.931 (95% CI: 0.864–0.998) relative to BC tissue which had an AUC of 0.864 (95% CI: 0.68–0.98) [11]. Zahran et al. (2021) focused on specific urinary metabolite signatures mainly, 8‐hydroxy‐20 deoxyguanosine (8‐OHdG), 1‐methyladenosine (1‐MA) and 1‐methylguanosine (1‐MG) of BC patients using the GC–MS platform. A combination of these markers with Cancer Antigen 15‐3 (CA15‐3, often used in breast cancer diagnosis), had AUC values ranging from 0.820 to 0.950 and diagnostic accuracy up to 95.5%, distinguishing early‐stage BC from healthy controls [12]. Considering all of these metabolomic studies, it is notable that they compared BC with healthy controls and so did not reflect the heterogeneity of breast malignancies of women attending clinics.

This current work uses direct infusion‐mass spectrometry (DI‐MS) to assess if the metabolomes of urinary, plasma and serum profiles could differentiate between BC and a wide range of control groups. DI‐MS offers several advantages over conventional analytical platforms such as GC–MS, LC–MS, and NMR, particularly in the dynamic range metabolites that can be detected but also speed, throughput, and analytical simplicity for complex samples. Its broad metabolite coverage and rapid analysis facilitate the detection of disease‐associated metabolic perturbations, thereby supporting investigations into disease pathogenesis and the discovery of potential biomarkers for early diagnosis [13, 14, 15].

In contrast to previous studies, this current study design incorporated control cohorts with benign breast pathologies. These included patients with benign breast disease (BBD) encompassing cysts, fibrocystic changes, papillomas and epithelial hyperplasia. Another control group consisted of symptomatic controls (SC) including breast‐related symptoms such as pain, nodularity, or palpable lumps but did not have a defined benign breast disease, and the final comparison was with healthy controls (HC). We aimed to ascertain if metabolomics could discriminate between these important clinical groups. The inclusion of these cohorts provided a more sensitive and clinically relevant framework for distinguishing BC‐specific metabolic alterations from those associated with common benign breast conditions, breast‐related symptoms as well as healthy controls. Examining urine, we observed metabolites in BC compared to each other experimental class which appeared more pronounced than with serum and plasma. Based on predicted identifications, the metabolites changes appeared to be linked to changes in amino acid and bioenergetic metabolism. If substantiated by future studies, these changes could be developed into novel diagnostic assays for BC that would not depend on substantial infrastructure. Furthermore, screening barriers linked to populations experiencing higher deprivation, lower socioeconomic status, and rural living conditions [16] may be overcome with easy‐to‐use and accessible assays.

## 2 Materials and Methods

A diagrammatic representation of the methodology to aid interpretation is provided in Figure S1.

### 2.1 Ethics Approval and Participant Recruitment

The study protocol (‘omic approaches to improve the diagnosis, management and treatment of breast cancer’; ‘BECA’) was ethically approved by Health Research Authority (HRA) and Health and Care Research Wales (HCRW) (IRAS Project ID: 306872; Protocol no: AU/IBERS/010; REC reference: 21/SC/0411; CPMS study ID: 54143). Recruits were adult females (age > 18 years old) presenting with some BC symptom(s) such as breast lumps and pain and attended the rapid access clinics at Glan Clwyd Hospital and Wrexham Maelor Hospital, Betsi Cadwaladr University Health Board (BCUHB) and Bronglais General Hospital, Hywel Dda University Health Board (HDUHB) from September 2022 to June 2024. The baseline demographic characteristics of the recruited participants, which include the information associated with BC risk factors, were collected (Table 1). BBD and HC displayed significant within‐group variation in age but no significant intergroup variation. The clinical and diagnostic features linked to the recruits are listed in Table 2. Data for medical history, family history of cancer, drug history, smoking status and BMI (body‐mass index) were collected. All available metadata are presented in Table S1.

**TABLE 1:** The baseline demographic characteristics of the participants (*n* = 400) in the study.

		Total	Sample groups				
			Breast cancer (BC)	Benign breast disease (BBD)	Symptom control (SC)	Healthy control (HC)	Between group significance *p*
*n*		400	118	148	95	39	< 0.05
Overall age range (Median ± SD)		19–92 (50 ± 15.94)	35–92 (64 ± 14.44)	21–84 (47 ± 13.57)	19–77 (43 ± 14.96)	22–79 (46 ± 14.69)	> 0.05
Age range (Median ± SD)	< 40	100 (25.64)	5 (4.35)	41 (28.08)	40 (43.48)	14 (37.84)	> 0.05
	40–49	92 (23.59)	24 (20.87)	43 (29.45)	19 (20.65)	6 (16.22)	> 0.05
	> 80	14 (3.59)	11 (9.57)	3 (2.05)	0	0	> 0.05
	Not stated	10	3	2	3	2	
Within group variation	*p*	< 0.05	< 0.05	> 0.05	< 0.05	> 0.05	
BMI (kg/m^2^) (Mean ± SD)		27.98 (±6.40)	28.92 (±6.64)	27.88 (±6.35)	27.37 (±6.20)	27.19 (±6.29)	> 0.05
	Current	58 (15.03)	13 (12.04)	24 (16.22)	19 (20.43)	2 (5.41)	< 0.05
Smoking habits, *n* (%) (*n* = 386)	Ex	115 (29.79)	41 (37.96)	44 (29.73)	25 (26.88)	5 (13.51)	< 0.05
	Never	213 (55.18)	54 (50.00)	80 (54.05)	49 (52.69)	30 (81.08)	< 0.05
Family history with BC, *n* (%)		142 (36.79)	43 (30.28)	57 (40.14)	38 (26.76)	4 (2.82)	< 0.05
Family history with other cancers, *n* (%)		138 (35.75)	45 (32.61)	46 (33.33)	36 (26.09)	11 (7.97)	< 0.05

Abbreviations: BMI, body mass index; NA, not applicable; SD, standard deviation.

**TABLE 2:** The clinicopathologic characteristics of breast cancer subjects (*n* = 118) in the study.

Clinicopathologic characteristics			Values
Breast cancer side, *n* = 118 (%)	Unilateral (*n* = 110)	Left	45 (38.14)
		Right	48 (40.68)
		Unknown	17 (14.41)
	Bilateral		4 (3.39)
	Unknown		4 (3.39)
Type, *n* = 118 (%)	Pre‐invasive		4 (3.39)
	Invasive		109 (92.37)
	Ductal carcinoma in situ (DCIS)		4 (3.39)
	Invasive ductal carcinoma (IDC)		58 (49.15)
	Invasive lobular carcinoma (ILC)		12 (10.17)
	Invasive mucinous carcinoma (IMC)		5 (4.24)
	Invasive tubular carcinoma (ITC)		1 (0.85)
	Invasive papillary carcinoma (IPC)		2 (1.69)
	Mixed carcinoma		5 (4.24)
	IDC accompanied DCIS		24 (20.34)
	Others/unknown		7 (5.93)
Subtypes, IBC; *n* = 114 (%)	Luminal A		77 (67.54)
	Luminal B		11 (9.65)
	HER2‐enriched		4 (3.51)
	Triple negative		6 (5.26)
	Unknown		16 (14.04)
Grade, IBC; *n* = 114 (%)	G1, *n* (%)		9 (7.89)
	G2, *n* (%)		50 (43.86)
	G3, *n* (%)		39 (34.21)
	Others/unknown		16 (14.04)
Tumour focality (*n* = 118)	Unifocal, *n* (%)		100 (84.75)
	Multifocal, *n* (%)		13 (11.02)
	Unknown, *n* (%)		5 (4.24)
Oestrogen (ER)	Positive		91 (82.73)
	Negative		10 (9.09)
	Unknown		9 (8.18)
Progesterone (PR)	Positive		73 (66.36)
	Negative		23 (20.91)
	Unknown		14 (12.73)
Human epidermal growth factor (HER2)	Positive		15 (13.64)
	Negative		81 (73.64)
	Unknown		14 (12.73)

Sampling occurred prior to any of the standard diagnostic procedures during their first clinical visit [17]. Samples were retrospectively classified as confirmed BC, BBD (linked to cysts, fibrocystic changes, papillomas and epithelial hyperplasia) or SC (based on exhibiting only painful breast symptoms). Healthy female volunteers, with no medical history of BC, were recruited at Bronglais General Hospital and Aberystwyth University (UK). All data were linked and transferred anonymised using a unique identifier before analysis. This study was conducted in accordance with the World Medical Association Declaration of Helsinki [18].

### 2.2 Sample Preparation and Extraction

The extraction of low molecular weight metabolites from urine, plasma and serum was based on Kirwan et al. and Marques et al. with modifications [19, 20].

Midstream 10–30 mL urine samples of 400 participants (BC = 118, BBD = 148, SC = 95 and HC = 39) were collected in a sterile container and immediately stored in −80°C for long‐term storage until further analysis. Blood samples were collected in serum‐separating yellow top (for serum) and ethylenediaminetetraacetic acid (EDTA) purple top (for plasma) vacutainers, respectively prior to centrifugation and transferred to cryovials which were stored in a −80°C freezer.

Aliquots of 1 mL urine sample were transferred to the microcentrifuge tubes and centrifuged (Microcentrifuge DS1524R, 220–230 V, 50 Hz, 500 W, Cat No: 9013111121, DLAB, China) at 6000×*g* for 5 min at 4°C. Samples of 300 μL were used for specific gravity measurement using the OPTI hand‐held refractometer (Ballingham Stanley, Serial #38‐01, UK) and normalised using distilled water to a common 1.006 specific gravity. A total of 40 μL of adjusted urine samples and 160 μL methanol/water (70:30, v/v) were transferred into the micro‐insert of HPLC glass vials for subsequent analysis. A quality control (QC) of pooled urine samples pool (‘master mix’) was also used in a separate vial, and another with 200 μL methanol/water (70:30, v/v) as the ‘blank’.

For plasma and serum samples (*n* = 52; BC = 33, BBD = 6, SC = 6, HC = 7), thawed aliquots were flash centrifuged at 6000×*g* and 200 μL transferred to a microcentrifuge tube and mixed with 1520 μL of HPLC grade methanol/chloroform mix (4:1 v/v). Following vortexing (G‐560E Vortex‐Genie 2, 240 V, 50 Hz, 0.5 Amps, Serial #2‐90073, Scientific Industries, USA), samples were placed in a shaker (Thermoshakers HCM100‐Pro, 100‐240 V, 50/60 Hz, 200 W, Cat No: 5062103100, DLAB, China) at 4°C for 15 min. Next, the samples were put in a −20°C freezer for 20 min prior to centrifugation at 21,000×*g*, 4°C for 5 min. Then, 100 μL samples were transferred to glass vials to be analysed. QC samples consisting of pooled plasma samples and pooled serum samples were generated, and solvent mix as ‘blank’.

### 2.3 Metabolite Profiling Using Direct Infusion‐Mass Spectrometry (DI‐MS)

Direct infusion‐mass spectrometry (DI‐MS) was performed using QEactive (Thermo Fisher Scientific, Bremen, Germany). Samples were run in batches of 150, with each batch having its own QC and blanks to allow for automatic correction. Data were acquired in positive and negative modes between *m/z* 70–1000 using a mass resolution of 140,000 (m/Δm at *m/z* 200). Samples (20 μL volume) were injected into a flow of 100 μL min^−1^ methanol: water (70:30, v/v). Ion intensities were acquired between *m/z* 50 and 1000 for 3.5 min at a resolution setting of 100,000 (at *m/z* 200), resulting in 3 (±1) ppm mass accuracy. Electrospray ionisation (ESI) source parameters were set according to manufacturer's recommendations.

Spectral binning approach used BinneR which also eliminated anomalous single scan *m/z* events, the average of spectra across the infusion profile. The modal accurate *m/z* was then extracted for each bin spectra and combined in a single intensity matrix (runs *x m/z*) for each ion mode [21]. Identifications consideration of isotope and different forms of ionisation based upon both a maximum error 5 ppm using the *mummichog* algorithm and also the BinneR algorithm [21] which was based on the rules described in Draper et al. [22] and assigned using MZedBD and public libraries, primarily KEGG (https://www.genome.jp/kegg/) and the human metabolome databases (https://hmdb.ca/) (accessed between May–September 2025). All isotopes/adducts were considered when deriving the identities for individual *m/z*. Metabolite identifications considered the following possible adducts: [M+]+, [M + H]+, [M + NH_4_]+, [M + Na]+, [M + K]+, [M‐NH_2_ + H]+, [M‐CO_2_H + H]+, [M‐H_2_O + H]+; [M−]−, [M − H]−, [M + Na − 2H]−, [M + Cl]−, [M + K − 2H]−. This level of identification conforms to MSI Level 2 and should be considered to be tentative. Where features were not corroborated across multiple adduct species, they were referred to only by their *m/z*.

Enrichment analysis was performed to identify the enriched metabolic pathways based on the Small Molecule Pathway Database (SMPDB), built into the MetaboAnalyst v6.0 package [23].

### 2.4 Statistical Analysis

MetaboAnalyst v6.0 package (accessed between May and September 2025) was utilised for univariate and multivariate analyses [23]. The data matrix was transformed using log_10_ transformation and Pareto scaling. Analysis of variance (ANOVA) and *t*‐test (corrected for false discovery rates [FDR correction (Benjamini–Hochberg)]) were performed to analyse between group and pair‐wise comparisons, respectively. Pairwise group comparisons used Tukey's HSD post hoc tests. Chemometric analysis used partial least squares‐discriminant analysis (PLS‐DA). The outputs were cross validated using leave one out cross validation (LOOCV) and model performance using Predicted Residual Sum of Squares (PRESS, from which *R* ^2^ is calculated) and the predictive power (*Q* ^2^).

### 2.5 Integrative Omics

The MixOmics package in R was used to relate metabolites identified in urine, plasma and serum samples. The design matrix of 0.6 representing the relationships between datasets was constructed based on the minimum pairwise correlation derived from the first component. Components explaining at least 70% of variances based on scree plot were selected as ncomp argument/parameter passed to the block.plsda() function for the Data Integration Analysis for Biomarker discovery using Latent variable approaches for Omics (DIABLO) analysis. The DIABLO algorithm was visualised using the plotDiablo() and network() functions to generate matrix scatter plot and network plot, illustrating sample distribution across components (latent variables) and positive and negative correlations between the metabolomic datasets respectively [24, 25].

## 3 Results

### 3.1 Urinary Metabolite Assessments by DI‐MS

**FIGURE 1:**
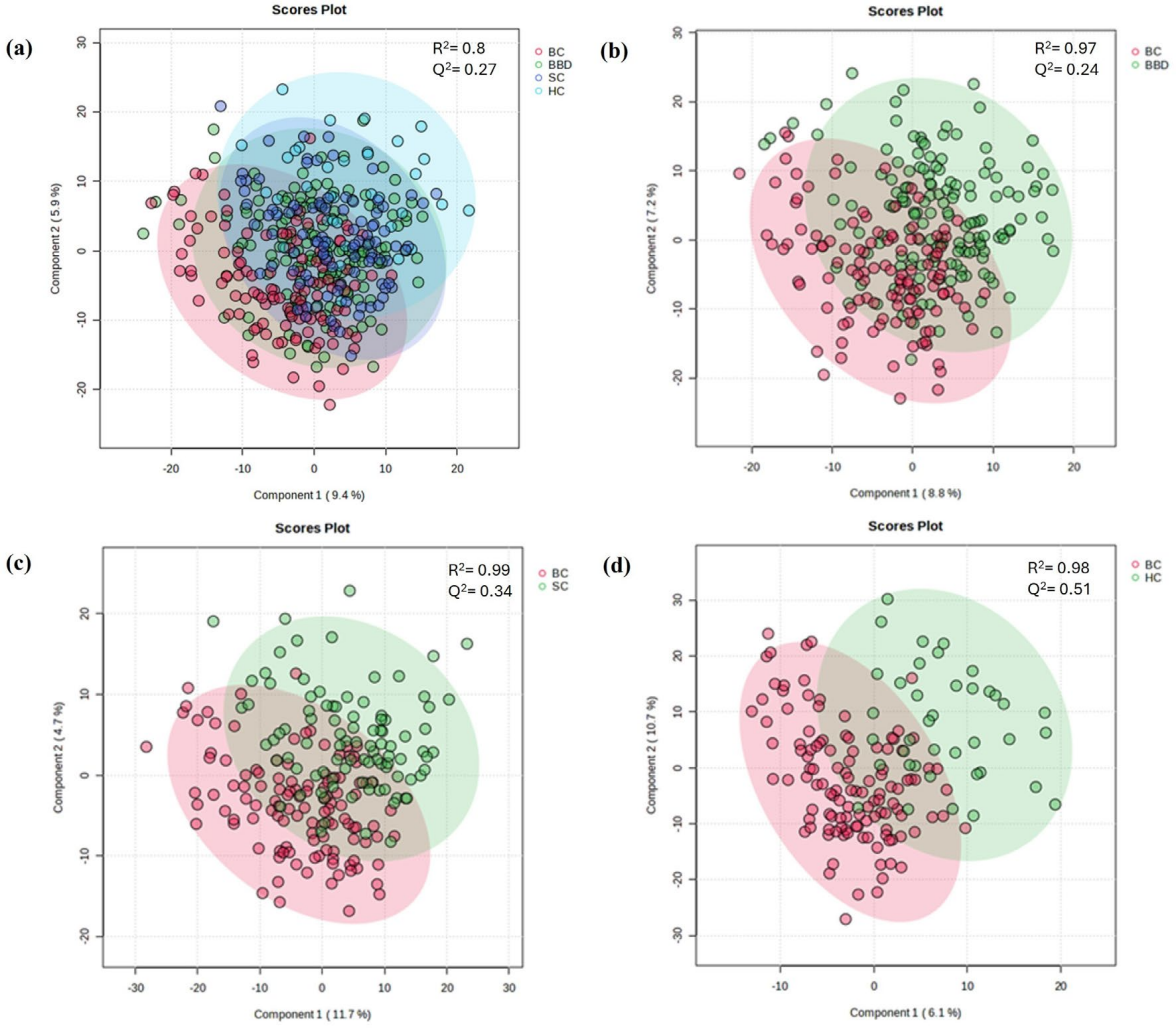
Supervised partial least squares–discriminant analysis (PLS‐DA) of urine metabolomes across four study groups and pairwise comparisons with breast cancer. PLS‐DA of urine metabolomes for (a) four study groups (*n* = 400); BC = breast cancer (*n* = 118), BBD = benign breast disease (*n* = 148), SC = symptom controls (*n* = 95), HC = healthy controls (*n* = 39). Pairwise PLS‐DA comparisons between BC and (b) BBD, (c) SC and (d) HC.

Untargeted metabolite profiling of urine samples from BC (*n* = 118), BBD (*n* = 148), SC (*n* = 95) and HC (*n* = 39) participants through DI‐MS detected 3644 *m/z* (2742 negative mode *m/z* and 902 positive mode *m/z*). Supervised PLS‐DA suggested that the metabolite profiles for BC, BBD, SC and HC groups showed considerable overlap (Figure 1a). In line with this, the PRESS/*R* ^2^ was high, and the predictive power (*Q* ^2^) was low. Some *m/z* had variable in projection (VIP) scores ≥ 1 in the PLS‐DA projection and these were tentatively identified (Figure S2). To further explore the data, pairwise comparisons were undertaken between BC and BBD (Figure 1b), SC (Figure 1c) and HC (Figure 1d). These suggested some drift in between the clusters, but the PRESS remained high although the *Q* ^2^ for the BC versus HC comparison was higher (Figure 1d). To assess if age could be a confounder in the analyses, profiles for different age groups in each experimental group were compared (Figure S2). This suggested that except with women in the oldest category (≥ 80 years old), age was not having a significant impact on any BC, BBD, SC and HC group differences.

Whilst the PLS‐DA suggested that the major sources of variation were not associated with the experimental classes, ANOVA was performed to identify any *m/z* that could distinguish between sample groups. A total of 189 significant *m/z* features (*p* < 0.05, corrected for false discovery rate [FDR]) differed between HC and the other experimental classifications. Pairwise comparisons using *t*‐test identified 121, 204 and 115 *m/z* features with FDR < 0.05 differentiating between BC and BBD, SC and HC, respectively.

**FIGURE 2:**
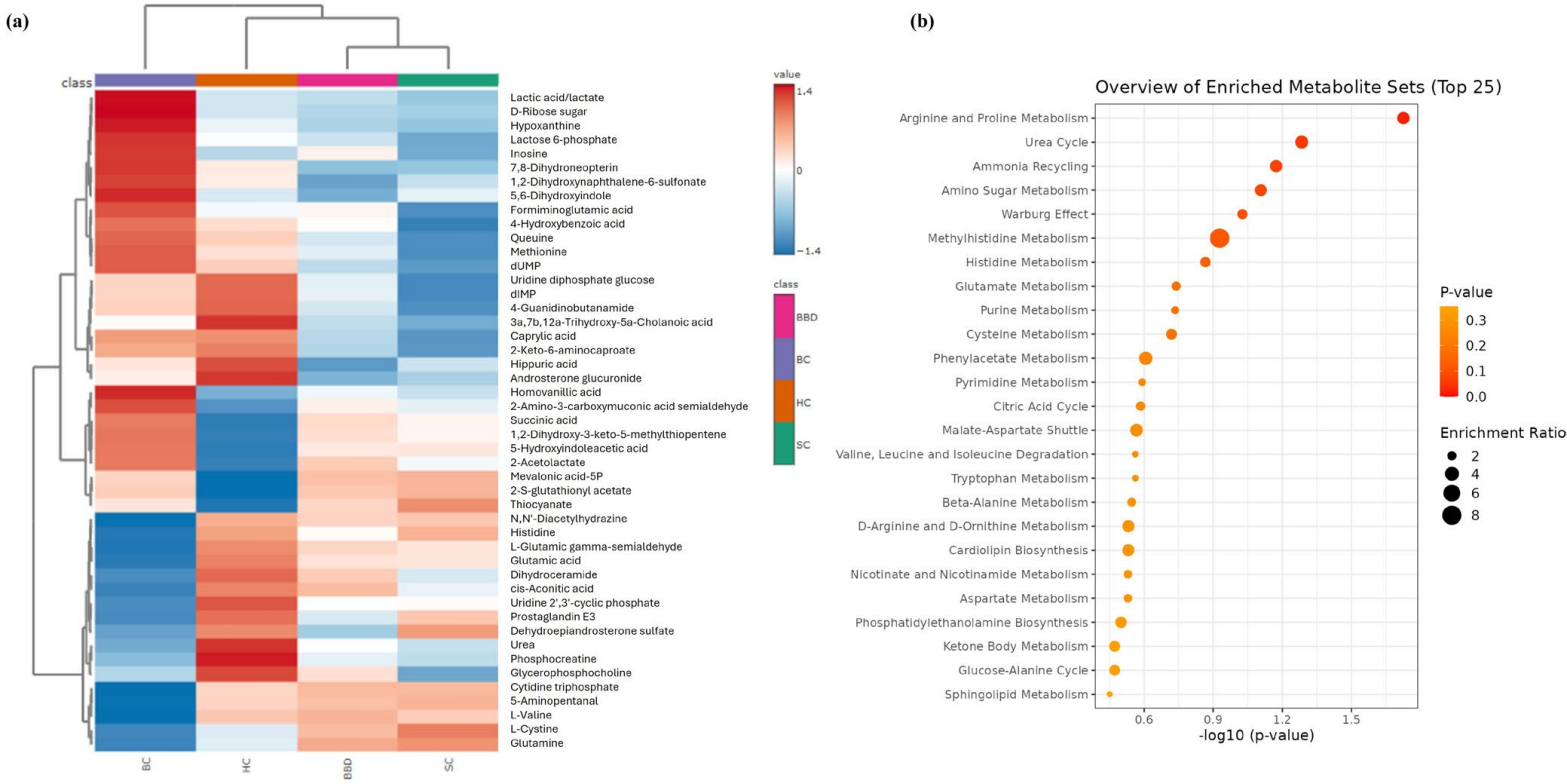
Metabolomic variation and pathway enrichment analysis across four urine study groups. (a) Heatmap of most significant sources of variation between four study groups (*n* = 400); BC = breast cancer (*n* = 118), BBD = benign breast disease (*n* = 148), SC = symptom controls (*n* = 95), HC = healthy controls (*n* = 39). (b) Pathway enhancement analyses based on mapping the most significant sources of variation on to human metabolic pathways (SMPDB).

The suggested differentially accumulating metabolites (DAM) were tentatively identified and associated with 47 metabolites (Table S3), whose accumulation patterns were compared in a heatmap (Figure 2a). DAMs that accumulated to higher levels in BC samples included n149.04573, n150.04909, p159.02783, p351.06772, n149.04318 and n197.04546 that were tentatively suggested to be lactic acid/lactate, D‐ribose sugar, hypoxanthine, lactose 6‐phosphate, 5,6‐dihydroxyindole and homovanillic acid, respectively. Conversely, *m/z* linked to glutamic acid, L‐glutamic gamma‐semialdehyde, histidine, urea and L‐valine were found to be at lower levels in the BC group while high in BBD, SC and HC groups (*p* < 0.05). The top sources of variation were also displayed using box and whisker plots (Figure S3). This suggested that, although the *R* ^2^/*Q* ^2^ values for PLS‐DA (Figure 1) were poor, some individual variables showed significant group specific differences.

Metabolite set enrichment analysis (MSEA) was performed to link the DAMs to discrete metabolic pathways (Figure 2b). A total of 25 pathways were enriched, with the most prominent being arginine and proline metabolism, urea cycle and ammonia recycling. This was consistent with possible shifts in amide and amino acid metabolism with BC.

### 3.2 Metabolites Profiling of Breast Cancer Clinical Characteristics

**FIGURE 3:**
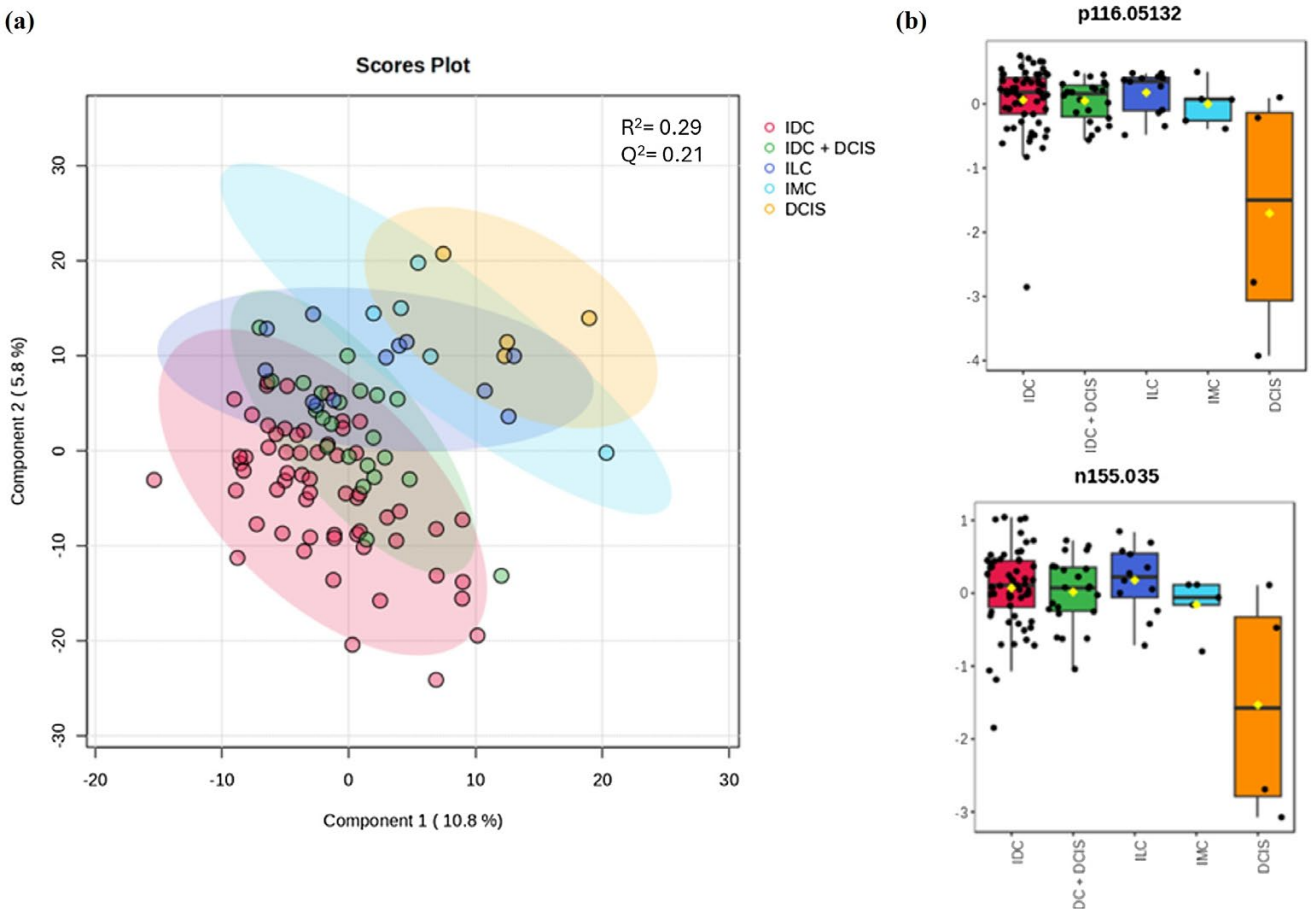
PLS‐DA of breast cancer histological subtypes and distinguishing urinary features. (a) PLS‐DA assessment of urine metabolite profiles from patients with Invasive Ductal Carcinoma (IDC, *n* = 58), IDC with Ductal Carcinoma In Situ (IDC + DCIS, *n* = 24), Invasive Lobular Carcinoma (ILC, *n* = 12), Invasive Mucinous Carcinoma (IMC, *n* = 5) and Ductal Carcinoma In Situ (DCIS, *n* = 4). (b) Boxes plot of *m/z* distinguishing DCIS and all other invasive breast cancer cases. *m/z* were targeted through ANOVA post hoc analysis applying Fisher's LSD. The n and p prefixes to the *m/z* refer to positive and negative ionizations, respectively.

We next studied changes in *m/z* levels within the BC subjects (*n* = 118) that could be linked with clinical characteristics (Table 2). Whilst the number of samples in each category differed considerably, this pilot level comparison was used to ascertain if this could have possibly contributed to the difficulty in separating between the groups as seen in Figure 1. BC types, particularly pre‐invasive mainly ductal carcinoma in situ (DCIS) and invasive BC (IBC), were suggested to have some distinct features based on the urinary metabolome. Although the PRESS (*R* ^2^) was high, PLS‐DA suggested separation between the DCIS and IBC samples mainly invasive ductal carcinoma (IDC) and IDC accompanied DCIS (Figure 3a). Identification of the major sources of variation using ANOVA (*p* = 0.05; correcting for FDR) suggested p116.05132 (tentatively identified as 2‐oxovaleric acid) and n155.035 (tentatively identified as orotate). Both were lower in DCIS cases (Figure 3b).

**FIGURE 4:**
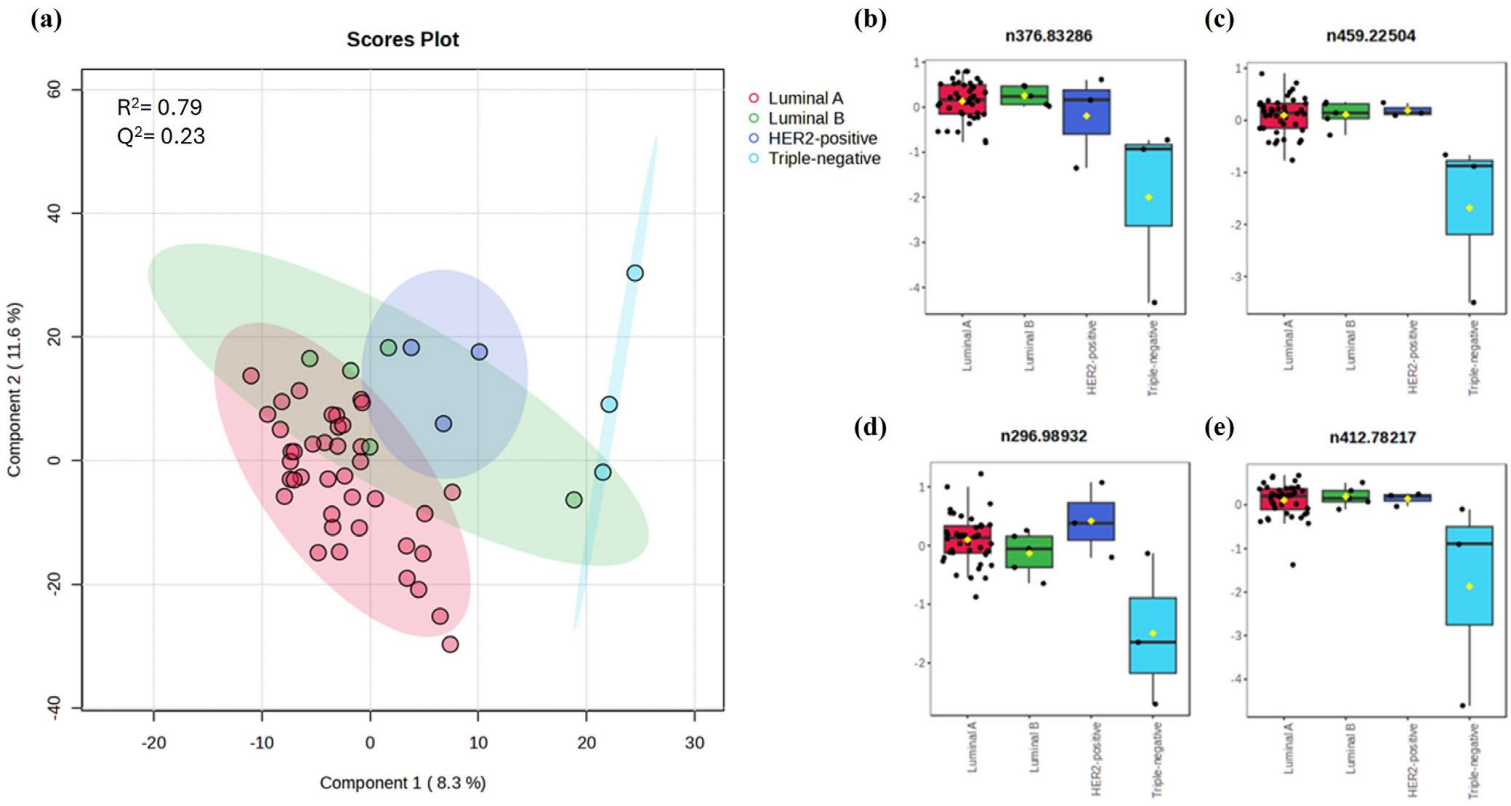
Metabolomic differentiation of breast cancer subtypes. (a) PLS‐DA assessment of urine metabolite profiles from patients with breast cancer types Luminal A (*n* = 77), Luminal B (*n* = 11), HER2‐positive (*n* = 3) and triple negative breast cancer (TNBC, *n* = 3). (b–e) Boxes plot of *m/z* distinguishing breast cancer types. *m/z* were targeted through ANOVA post hoc analysis applying Fisher's LSD. The n prefixes to the *m/z* refers to negative ionisation.

Considering variation based on clinical subtypes, the PLS‐DA score showed the greatest variation plot with triple negative breast cancer (TNBC, ER‐ PR‐ HER2‐), a high‐grade BC and most aggressive form subtype with relatively poor prognosis (Figure 4). This formed a cluster away from the other subtypes (Luminal A, B and HER2‐positive) although the PLD‐DA model had a high *R* ^2^ and a low *Q* ^2^. This notwithstanding, ANOVA (*p* < 0.05, corrected for FDR) revealed four *m/z* which were significantly lower in TNBC cases (Figure 4b–d). The box plots indicated the value of the univariate statistical comparisons, even when PLS‐DA results could be suggesting over‐fitting. The *m/z* were n296.98932, n376.8328, n412.78217, with a tentative identification only possible for n459.22504 (a monoacylglyceride (0:0/20:5/0:0)).

### 3.3 Blood (Plasma and Serum) Metabolomic Assessments

**FIGURE 5:**
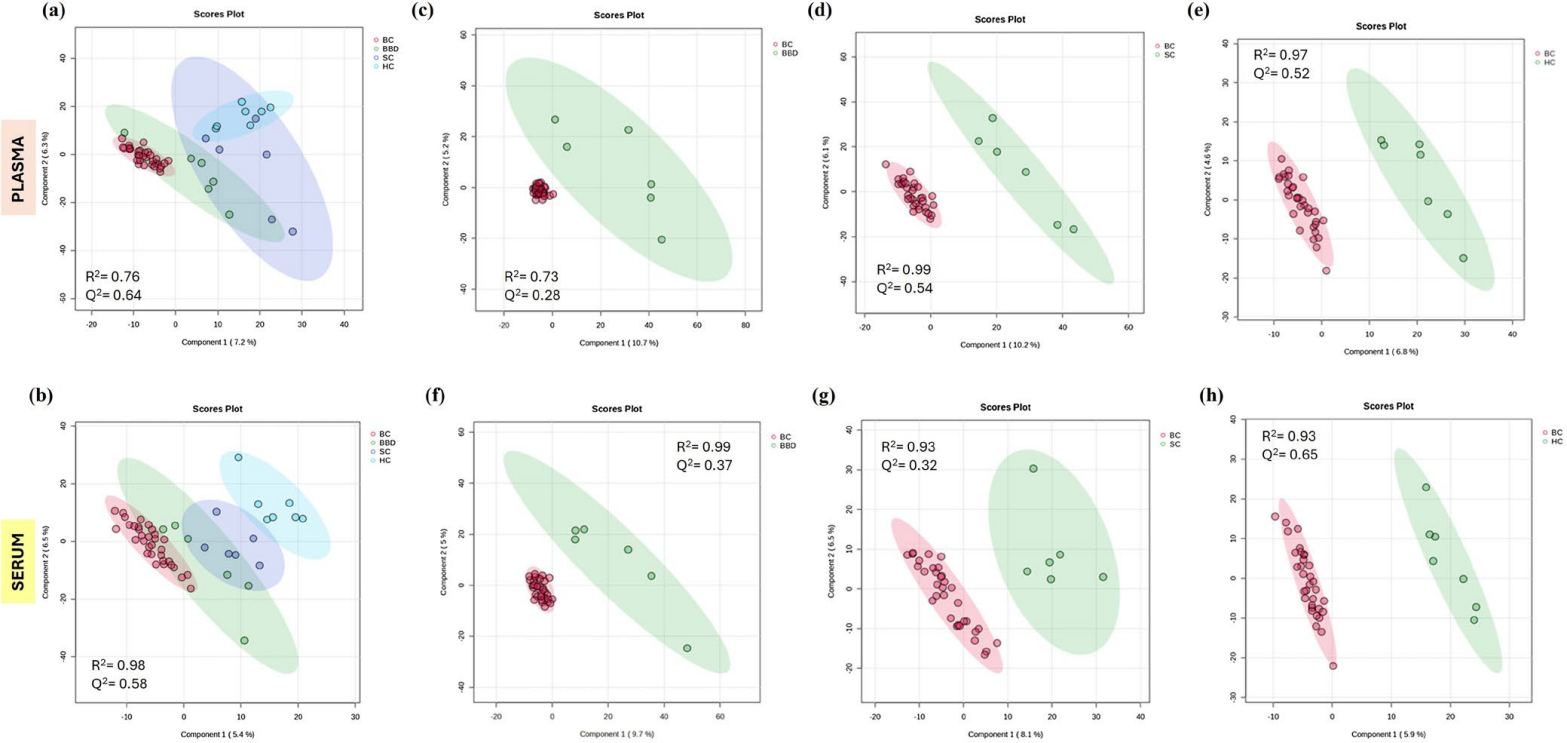
PLS‐DA of plasma and serum metabolomes in breast cancer and control groups. Supervised partial least squared‐discriminant analysis (PLS‐DA) of (a) plasma and (b) serum metabolomes for BC = breast cancer (*n* = 33), BBD = benign breast disease (*n* = 6), SC = symptom controls (*n* = 6) and HC = healthy controls (*n* = 7). Pairwise PLS‐DA comparisons of plasma BC against (c) BBD, (e) SC and (g) HC and sera of BC against (d) BBD, (f) SC and (h) HC samples.

**FIGURE 6:**
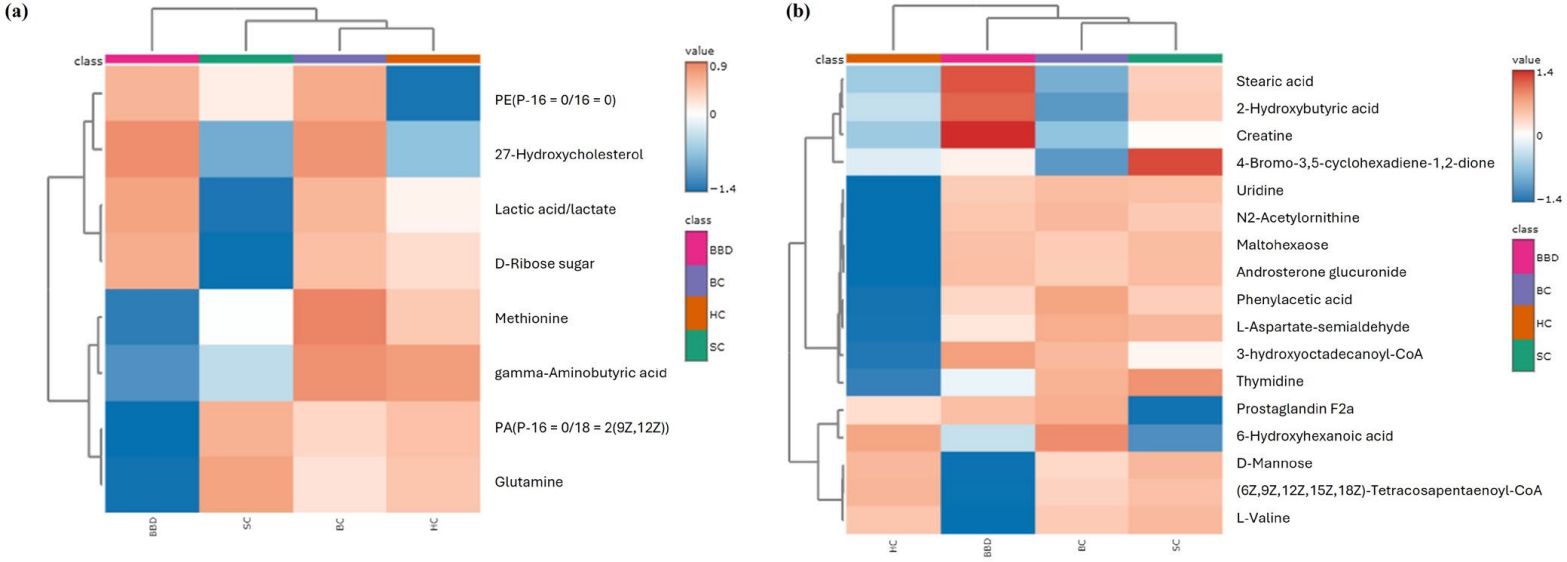
Metabolic variation heatmaps for plasma and serum across clinical groups. The most significant sources of variation between BC = Breast Cancer (*n* = 33), BBD = Benign Breast Disease (*n* = 6), SC = Symptom control (*n* = 6) and HC = Healthy control (*n* = 7) in (a) plasma and (b) serum metabolite profiles.

Our BECA sample collection, includes serum and plasma (*n* = 52) obtained from the same patient cohort used for urine assessments. Although the sample size was smaller, these samples were profiled as a pilot study to investigate if they showed group specific variation. PLS‐DA score plots indicated distinct clusters of BC from BBD, SC and HC group with minimal overlap based on the plasma (Figure 5a) and serum metabolome (Figure 5b) profiles. However, given the limited class sizes, these findings should be interpreted with caution and require validation in larger cohorts. The major sources of variation in plasma and serum samples were targeted by *t*‐test (*p* < 0.05, correcting for FDR) and their pairwise comparisons as depicted in Figure 5c–h. The targeted *m/z* were tentatively identified and presented as heatmaps and demonstrated metabolite shifts across sample groups (Figure 6a,b). In plasma, metabolites that were relatively higher levels in BC included the *m/z* tentatively identified as methionine (p172.0403) and gamma‐aminobutyric acid (p126.05268). In serum, 6‐hydroxyhexanoic acid (n131.07144) and phenylacetic acid (p91.05437) were elevated while hydroxybutyric acid (n163.06136), creatine (n112.05165), stearic acid (n329.27014) and bromo‐cyclohexadiene‐dione (n264.85065) were lower in serum samples of BC subjects relative to the other cohorts.

**FIGURE 7:**
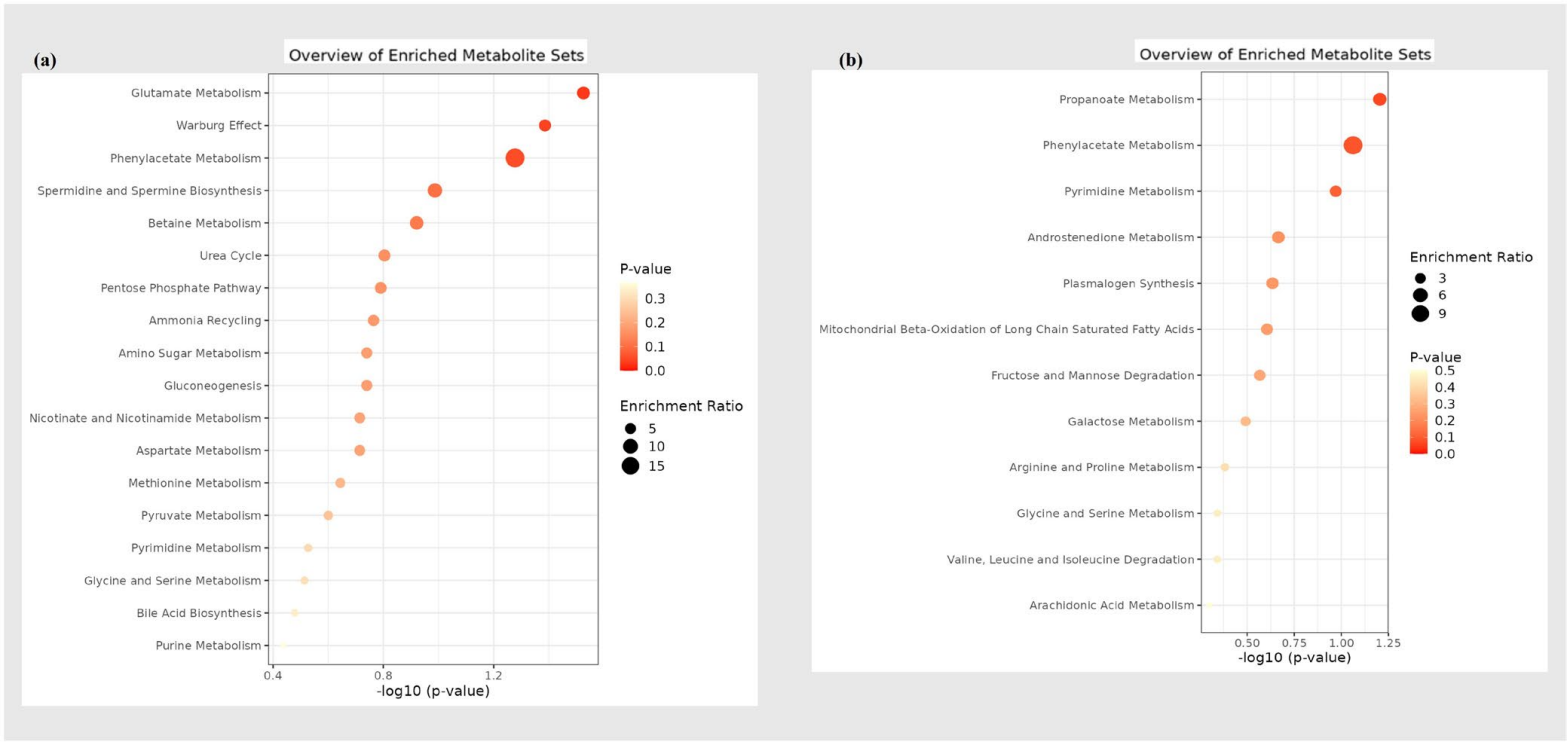
Pathway Enrichment in Plasma and Serum Metabolomes. Enrichment analysis of key metabolites discriminating between urine from breast cancer, benign breast disease, symptomatic and healthy control samples demonstrating the highly altered metabolisms in (a) plasma and (b) serum. Enrichment ratio = the dimension of the bubble represents the enrichment level.

Targeted metabolites from plasma and serum were mapped to KEGG pathways (Figure 7a,b, respectively). In plasma, glutamate metabolism, Warburg effect, and phenylacetate metabolism were significantly enriched (*p* < 0.05). With serum, propanoate metabolism was significantly targeted (*p* < 0.05), with hit metabolites including 2‐hydroxybutyric acid (n163.06136).

### 3.4 Liquid Sample Metabolome Comparisons

**FIGURE 8:**
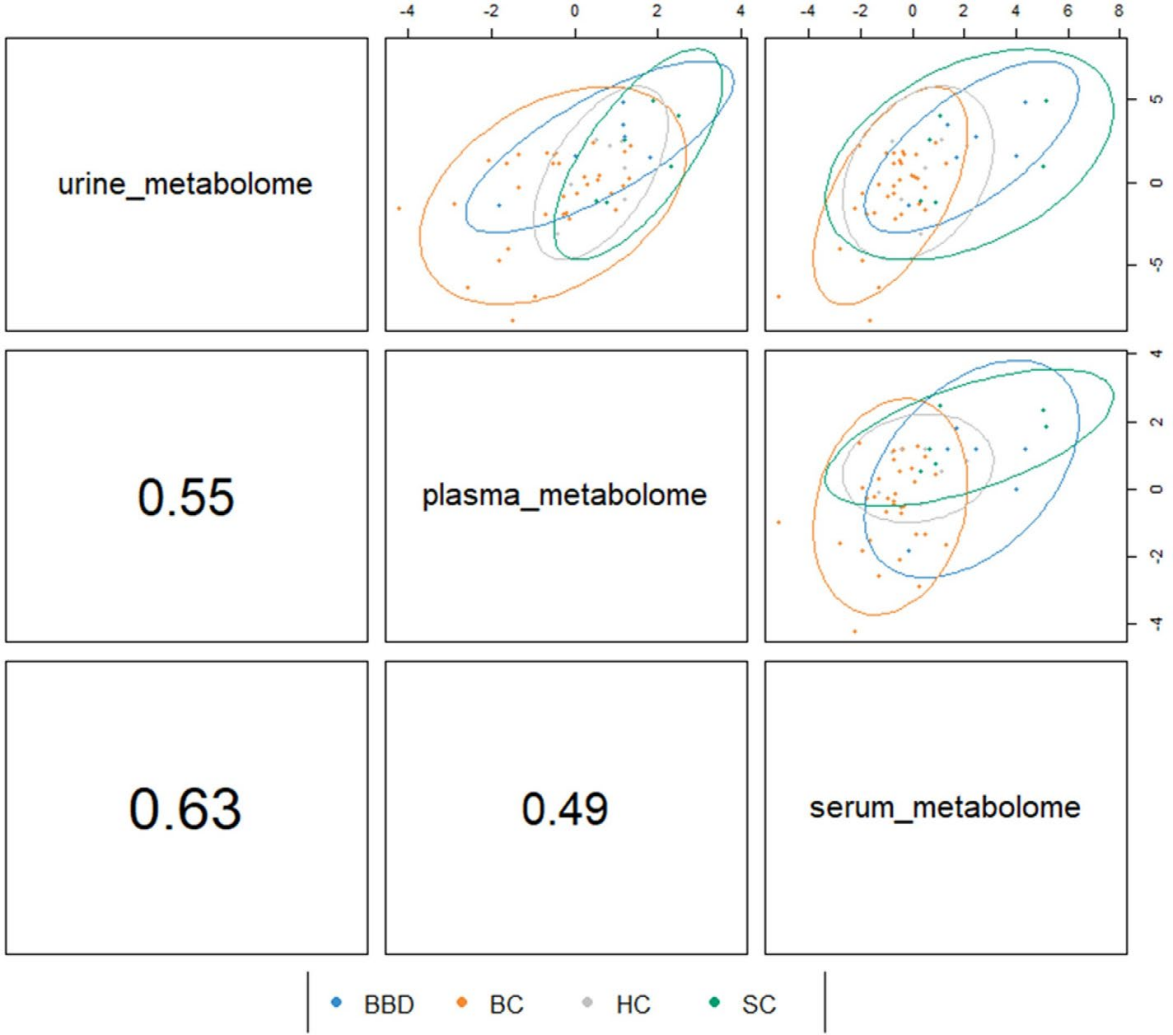
DIABLO Integration of Urine, Plasma, and Serum Metabolomes. Correlating metabolites of urine (‘urine_metabolome’), plasma (‘plasma_metabolome’) and serum (‘serum_metabolome’) discriminating between BC = Breast Cancer (orange), BBD = Benign Breast Disease (blue), SC = Symptom control (green) and HC = Healthy control (grey) groups using plotDiablo() function in MixOmics package with design matrix 0.6 and eight principal components.

The DIABLO algorithm was applied to investigate correlated significant (*p* < 0.05) metabolite changes in urine, plasma and serum that discriminated between BC (*n* = 33), BBD (*n* = 6), SC (*n* = 6) and HC (*n* = 7) sample groups. Although the sample sizes are small, DIABLO is specifically designed for high‐dimensional, low‐sample‐size settings and incorporates dimensionality reduction and sparsity constraints to mitigate overfitting [25]. Given the difference in numbers in each group, in some cases quite small, the following relationships should be considered to be suggestive, not definitive. The matrix scatter plot illustrated pairwise DIABLO (block PLS) sample correlation based on the first component of each dataset (Figure 8). Samples were colour‐coded by group (BC: orange, BBD: blue, SC: green and HC: grey) displayed moderate separation, with 95% confidence ellipses representing group level clustering. Pairwise correlation coefficients between datasets were 0.55 for urine and plasma metabolites, 0.63 for urine and serum metabolites while 0.49 for plasma and serum metabolites. This suggested that there was some moderate correlation in metabolite changes across the datasets.

**FIGURE 9:**
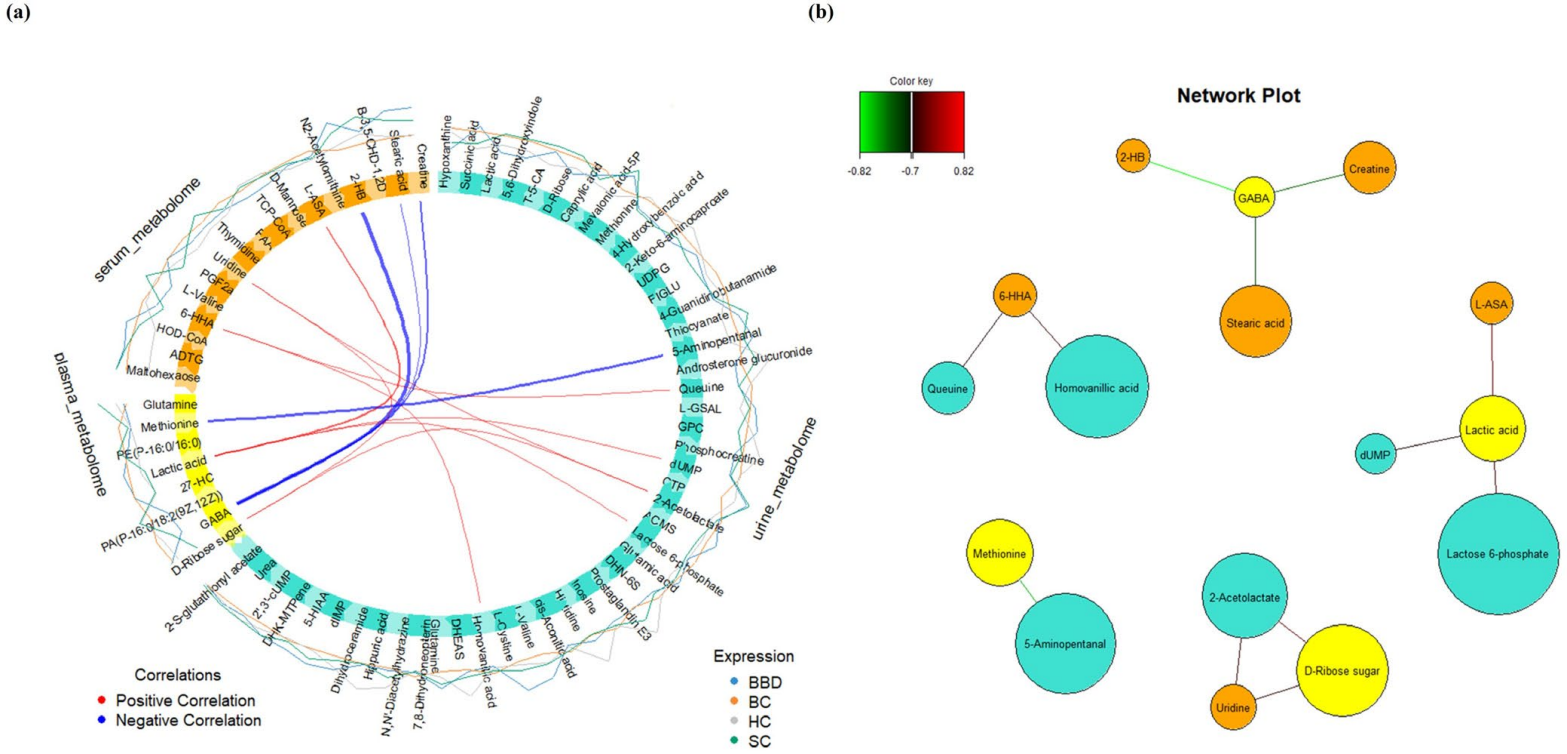
CIRCOS and network mapping of multi‐fluid metabolites across clinical groups (a) CIRCOS plot and (b) Network visualisation of key metabolites of urine (urine_metabolome; blue), plasma (plasma_metabolome; yellow) and serum (serum_metabolome; orange) discriminating between BC = Breast Cancer, BBD = Benign Breast Disease, SC = Symptom control and HC = Healthy control groups, using network() function in MixOmics package with an absolute cutoff value of 0.7. Key: 2′,3′‐cUMP = Uridine 2′,3′‐cyclic phosphate; 27‐HC = 27‐Hydroxycholesterol, 2‐HB = 2‐Hydroxybutyric acid, 5‐HIAA = 5‐Hydroxyindoleacetic acid, 6‐HHA = 6‐Hydroxyhexanoic acid, ACMS = 2‐Amino‐3‐carboxymuconic acid semialdehyde, ADTG = Androsterone glucuronide, ASA = L‐Aspartate‐semialdehyde, B‐3,5‐CHD‐1,2D = 4‐Bromo‐3,5‐cyclohexadiene‐1,2‐dione, CTP = Cytidine triphosphate, DH‐3‐K‐MTPene = 1,2‐Dihydroxy‐3‐keto‐5‐methylthiopentene, DHEAS = Dehydroepiandrosterone sulphate. DHN‐6S = 1,2‐Dihydroxynaphthalene‐6‐sulfonate, FIGLU = Formiminoglutamic acid, GABA = gamma‐Aminobutyric acid, GPC = Glycerophosphocholine, HOD‐CoA = 3‐hydroxyoctadecanoyl‐CoA, L‐GSAL = L‐Glutamic gamma‐semialdehyde, PAA = Phenylacetic acid, PGF2a = Prostaglandin F2a, T‐5‐CA = 3a,7b,12a‐Trihydroxy‐5a‐Cholanoic acid, TCP‐CoA = (6Z,9Z,12Z,15Z,18Z)‐Tetracosapentaenoyl‐CoA, UDPG = Uridine diphosphate glucose.

The relationships (positive or negative) between key tentatively identified metabolite features across urine, serum and plasma datasets were mapped and depicted in a network plot (Figure 9). Links between such as glutamine, methionine, lactic acid/lactate and D‐ribose sugar were suggested in urine and plasma samples, whereas androsterone glucuronide and L‐valine were targeted in both urine and serum samples. Plasma lactic acid/lactate showed positive correlations with urinary lactose 6‐phosphate and dUMP and serum L‐aspartate‐semialdehyde suggesting that these metabolites exhibited similar accumulation patterns. Furthermore, plasma D‐ribose sugar also positively correlated with serum uridine and urinary 2‐acetolactate. Conversely, plasma gamma‐aminobutyric acid (GABA) displayed negative correlations with serum stearic acid, creatine and 2‐hydroxybutyric acid reflecting opposing expression patterns of these linked metabolites. In particular, elevated plasma GABA level coincided with reduced serum stearic acid, creatine and 2‐hydroxybutyric acid levels in BC subjects.

## 4 Discussion

BC is a multifactorial and heterogenous disease that possesses specific metabolic characteristics [26, 27]. One of the fundamental features of cancer cells that distinguish them from normal cells is the metabolic alterations in glucose, amino acids and fatty acids metabolism, reflecting BC‐linked changes in energy and signalling to support rapid growth and metastasis [28, 29, 30, 31, 32, 33]. Therefore, metabolomics provides a general snapshot of the dysregulated metabolic processes and could yield potentially diagnostic biomarkers for BC [34, 35].

Urine metabolites have been explored as alternative sources for biomarker reservoirs as diagnostic tools to ameliorate early detection and treatment outcomes for BC [9, 24, 34, 35, 36, 37]. However, few studies focus on the differences in urine changes between BC and breast abnormalities such as BBD, or symptoms but often use healthy women as controls. The present study utilised DI‐MS to acquire the metabolite profile of BC subjects and compared it with the women presented with BBD and controls (SC and HC). We used multivariate approaches and ANOVA corrected for FDR, but assessments should be improved by machine learning‐based approaches in the future. Further, whilst DI‐MS can supply only tentative identifications of metabolite changes, it is a highly sensitive approach that can capture changes in the metabolome [22, 38, 39]. Further, by considering multiple adducts/isotopes of a given metabolite in our high‐resolution (< 5 ppm) *m/z* data, greater confidence in the identifications can be provided. Using DI‐MS, urinary metabolites could distinguish between BC patients and those presented with BBD (e.g., fibroadenoma, cysts and fat necrosis), symptomatic (e.g., breast pain, nodularity or palpable lumps), and HC groups.

Accepting the caveat that our identifications require follow on confirmation, we observed changes that were supported by the wider literature. We observed that urinary glutamine and glutamic acid were lower in BC patients compared to other groups. Slightly similar higher levels of plasma glutamine and serum L‐valine were observed in BC, SC and HC, while significantly reduced in BBD which potentially reflects the shift within benign changes. Others have reported lower glutamine, L‐lysine and L‐valine concentrations in BC (*n* = 91) than HC (*n* = 20) plasma [40, 41]. In a cell line based study, glutamine and branched chain amino acids (BCAAs) including leucine, isoleucine and valine were utilised by the BC cells while cysteine and glutamic acid were significantly excreted [42].

Our observed increases in glutamine could influence tumour progression, immune function, and the tumour microenvironment [43, 44, 45]. Glutamine supports BC cell proliferation by providing essential carbon and nitrogen for macromolecular biosynthesis and by fuelling the tricarboxylic acid (TCA) cycle, rendering many tumour cells highly glutamine‐dependent. This glutamine‐centred metabolic reprogramming can also suppress antitumor immunity, in part by restricting glutamine availability to immune cells and altering immune regulatory pathways [46]. Moreover, reductive glutamine metabolism feeds into the rewired TCA flux associated with the Warburg effect, where aerobic glycolysis and lactate production are favoured despite adequate oxygen. Elevated lactate (as also seen in our study, Figure S4) further promotes immune evasion and tumour progression, partly through aberrant protein lactylation, which has been shown to drive immunosuppression and microenvironmental reprogramming [42]. Glutamate/glutamine effects can be linked to the mammalian target of rapamycin (mTOR) pathway, which has been identified as a vital pathway in cancer growth [42]. When amino acid metabolism is reprogrammed, glutamine and BCAAs were involved in the regulation of the mTOR pathway as the signalling nutrients that activate the pathway, linking metabolism and immunity as well as affecting the nutritional survival of immune cells in the TME [29, 44]. Competition for glutamine between tumour and immune cells in the TME may cause immune cell dysfunction, as cancer cells have a higher demand for glutamine [47].

A further aspect of altered nitrogen metabolism was suggested by the tentatively identified metabolites including the lower levels of urea in BC while homovanillic acid and 5‐hydroxyindoleacetic acid were higher, when compared to BBD, SC and HC. In contrast, Nam et al. observed higher urea levels in BC, whereas homovanillate and 5‐hydroxyindoleacetate were lower compared to HC (*p* < 0.05) in urine. This pattern could be linked to race/ethnicity (Asian‐Korean) and histological types of BC (IDC, ILC and DCIS) [40, 48]. For example, 117 and 26 plasma metabolites were identified to be significantly different between races (African American and Caucasian American females) and subtypes (ER+/PR+ and TNBC), respectively [40]. Although, ethnicity data were not gathered in our clinical data, it is probable that most of our recruits were of white European ancestry/culture (e.g., diet), and this could have influenced our observations.

D‐ribose sugar was also significantly elevated in the urine samples of the BC group. D‐ribose, a 5‐carbon sugar, is essential in cell resynthesizing [49, 50]. High levels of D‐ribose were increased in urine and blood of type II diabetes mellitus patients, which may trigger tissue inflammation [50]. Therefore, high concentrations of D‐ribose in urine may probably result in elevated reactive oxygen species (ROS), leading to inflammation and malignancy in the BC group [51]. Furthermore, high ROS levels upregulate p53 to activate the TP53 inducible glycolysis and apoptosis regulator (TIGAR) and indirectly inhibit phosphofructokinase‐1 (PFK1) to shift glycolytic flux towards the pentose phosphate pathway (PPP). Prolonged H_2_O_2_‐induced oxidative stress enhanced growth and survival of MCF‐7 cells, suggesting chronic ROS exposure may increase their tumorigenic and metastatic potential [52]. Many tumours exhibit a proinflammatory microenvironment where infiltrated immune cells release factors fostering the differentiation of normal cells and facilitating the survival and metastasis of cancer cells [52]. Interestingly, the tentatively identified d‐ribose sugar, lactic acid/lactate, were elevated while glutamine, glutamic acid, lysine and histidine were decreased in TNBC relative to Luminal A/B and HER2‐enriched groups. We also observed that histidine, glutamine, lactic acid/lactate and d‐ribose sugar levels were lower in the urine of DCIS patients compared to invasive BC patients. These observations align with the wider literature, which suggests that histidine contributes to BC inflammation through histamine‐mediated signalling that affects tumour growth and treatment response [52]. Nevertheless, due to the small sample size of BC subjects examined, these observations should only be considered to be suggestive, and validation in a larger independent cohort is required.

A different metabolomic change in BC is suggested from the shift of several nonpolar metabolite accumulations across sample groups were observed. This may suggest alterations in lipid metabolism and steroidogenesis associated with breast tumorigenesis [53, 54, 55]. For instance, the tentatively identified metabolites, particularly dihydroceramide and dehydroepiandrosterone sulphate (DHEA sulphate), levels were lower in urine of BC compared to other cohorts. Dihydroceramides are intermediates in ceramide biosynthesis and bioactive sphingolipid metabolites involved in cell cycle arrest, autophagy and apoptosis [56]. The reduction in urinary dihydroceramide levels in the BC group may suggest dysregulated sphingolipid metabolism, which potentially contributes to the survival and progression of breast tumour [57]. DHEA‐sulphate is a potential predictor of BC increased risk among premenopausal [55] and also postmenopausal women (particularly ER‐positive patients [OR 1.09, 95% CI 1.03–1.16]) [58]. Conversely, DHEA‐sulphate can inhibit key processes involved in BC metastasis, including cell migration and invasion, in breast tumour cell lines [59]. Targeting lipid changes in BC could be important in new therapeutic strategies, which could be based on lipophilic drugs [60].

Our integrative analysis suggested correlations between urine, serum and plasma signatures suggesting systemic metabolic reprogramming which included an upregulation of glycolytic flux, nucleotide biosynthesis and the PPP. The positive associations such as between plasma lactic acid/lactate and urinary lactose‐6‐phosphate, dUMP and serum L‐aspartate‐semialdehyde are consistent with the Warburg effect [61, 62]. The concomitant association with metabolites such as dUMP and L‐aspartate‐semialdehyde could reflect enhanced flux through pyrimidine and aspartate pathways, both of which are essential for nucleotide synthesis and tumour proliferation [62, 63, 64, 65]. Similarly, the positive correlation of plasma D‐ribose with serum uridine and urinary 2‐acetolactate could support both nucleotide biosynthesis and nicotinamide adenine dinucleotide phosphate (NADPH) production, vital for sustaining redox balance and cancer cell growth [61, 66, 67]. Conversely, the negative correlations observed between plasma GABA and serum metabolites including stearic acid, creatine and 2‐hydroxybutyric acid suggest a metabolic trade‐off that characterises BC progression. Elevated plasma GABA coincides with diminished lipid availability, altered energy buffering and disrupted redox intermediates. Reduced serum stearic acid may reflect perturbed fatty acid homeostasis and membrane remodelling, processes implicated in metastasis and tumour aggressiveness [68]. The reduction of creatine points to compromised energy storage dynamics and decreased 2‐hydroxybutyric acid may indicate shifts in oxidative stress and glutathione‐related metabolism.

**FIGURE 10:**
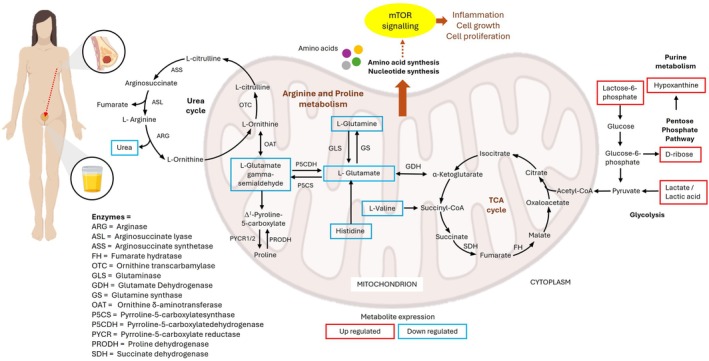
Schematic diagram highlighting urinary metabolite changes linked to breast cancer as suggested in this study. Metabolically, arginine and proline metabolism was significantly dysregulated/enriched (*p* < 0.05). The downregulation of glutamine and its intermediates likely reflects their rapid consumption by cancer cells to support uncontrolled growth. As the most abundant circulating amino acid, glutamine fuels tumour progression by providing carbon and nitrogen through glutaminolysis, aiding energy production, reducing oxidative stress, preserving mitochondrial function and sustaining proliferating cells [40, 41, 42, 43, 67]. The up‐regulation of metabolites like D‐ribose and proline intermediates (via the PPP and proline biosynthesis) reflects increased oxidative stress and energy demands of rapidly proliferating cancer cells [65]. Glutamate/glutamine effects can be linked to the mammalian target of rapamycin (mTOR) pathway, which has been identified as a vital pathway in cancer growth [40]. When amino acid metabolism is reprogrammed, glutamine and BCAAs were involved in the regulation of the mTOR pathway as the signalling nutrients that activate the pathway, linking metabolism and immunity as well as affecting the nutritional survival of immune cells in the TME [22, 42]. Furthermore, the alterations in mTOR play a key role in tumorigenesis and metastasis in BC, promoting growth by regulating glycolysis, angiogenesis, growth factor pathways, lipid metabolism and autophagy [67]. Figure partially created using BioRender.

Although based on tentative identifications, our metabolomic assessments of liquid biomarkers were mostly consistent with the wider literature and suggest that breast malignancy is potentially driven by multiple metabolic dysregulations, inflammatory responses, cell growth and proliferation. Figure 10 illustrates a proposed mechanistic framework linking urinary metabolite signatures with breast malignancy. We have included a speculative link to the activation of mTOR signalling as this plays a key role in tumorigenesis, metastasis and drug resistance in BC, promoting growth by regulating glycolysis, angiogenesis, growth factor pathways, lipid metabolism and autophagy [69]. Persistent activation of mTOR in normal cells leads to increased metabolic activity and cell proliferation, extends lifespan, and can even lead to cellular immortalisation, thereby contributing directly or indirectly to malignancy [51, 70]. These interpretations are derived based on *inferred associations* rather than direct evidence; additional studies investigating the association of these metabolite signatures are warranted to substantiate and refine the current findings. This could involve the association of metabolites with single nucleotide polymorphisms linked to relevant genes or the use of animal BC models or cell lines. Further, the changes seen with different BC subtypes should be substantiated with sampling larger patient cohorts.

While this study demonstrates the feasibility and potential of a urine‐based metabolomics approach for BC detection and suggests novel biological insights, several limitations should be acknowledged to contextualise the findings and guide future improvements. Although the study included relatively large cohorts compared with similar discovery studies based on urine samples, the sample size for each cohort remained modest and uneven, which may limit statistical power and generalisability as well as robustness of the observation. Therefore, future study with larger, multi‐centre cohorts is required to strengthen subgroup analyses and external validation [71]. There were also potential confounding factors that may have influenced the urinary omics profiles, including differences in age distribution across cohorts, particularly the higher median age observed in the BC group relative to the other groups. However, assessment of metabolomic profiles across age categories suggested that, except for women aged ≥ 80 years, age did not significantly influence differences between the BC, BBD, SC and HC groups. Similar, BMI and smoking status did not appear to be a significant source of variation in the metabolite profiles. However, additional factors such as diet, kidney function, systemic inflammation, fasting status, and hormonal or menopausal status may also have contributed to metabolic variability and were not considered. To reduce some of these effects, future validation studies should aim to standardise cohort demographics and sample collection protocols, while incorporating additional clinical parameters such as C‐reactive protein (CRP) to quantify inflammatory status as well as evaluate potential confounding factors to better account for biological variability. Additionally, although urinary concentration variability was normalised using specific gravity, complementary normalisation measures, including urinary creatinine, could be incorporated to further evaluate the robustness and consistency of normalisation approaches across studies [72].

Nevertheless, these variables were intentionally not tightly controlled in the present study as the primary aim was to identify urinary biomarkers suitable for translation into a clinically applicable lateral flow assay under real‐world conditions. Consequently, the study design sought to capture biologically and clinically realistic variability within the patient population. Addressing these methodological and biological limitations in future studies will further enhance the robustness, reproducibility, and translational potential of urine‐based multi‐omics approaches for BC detection.

## Author Contributions

**Nur Aimi Aliah Zainurin:** formal analysis (lead), methodology (equal), project administration (equal), writing – original draft (lead), writing – review and editing (supporting). **Anuradha U. K. H. Bambarandhage:** formal analysis (supporting), writing – review and editing (supporting). **Michelle Moreno Escalona:** formal analysis (supporting), writing – review and editing (supporting). **Dimitra Ivanova:** formal analysis (supporting). **Tim Gate:** resources (supporting), writing – review and editing (supporting). **Helen Tench:** resources (supporting), writing – review and editing (supporting). **Manfred Beckman:** formal analysis (supporting), resources (supporting), writing – review and editing (supporting). **Helen Phillips:** formal analysis (supporting), supervision (supporting), writing – review and editing (equal). **Mandana Pennick:** conceptualization (supporting), project administration (supporting), resources (supporting), supervision (supporting), writing – review and editing (supporting). **Luis A. J. Mur:** conceptualization (lead), formal analysis (supporting), funding acquisition (lead), project administration (lead), supervision (supporting), writing – review and editing (lead).

## Funding

N.A.A.Z. was supported by a Graduate Excellence Programme (GrEP), Majlis Amanah Rakyat (MARA) fellowship (Grant No. 330408376064). L.A.J.M. is partially supported by Shandong Province ‘Double‐Hundred Talent Plan’ Teams (Grant No. WSR2023049). The authors would like to acknowledge the Institute of Biomedical Sciences (IBMS) for the research grant award.

## Ethics Statement

The study was reviewed and approved by the Health Research Authority (HRA) and Health and Care Research Wales (HCRW). Registry and the Registration No. of the Study: IRAS Project ID: 306872; Protocol no: AU/IBERS/010; REC reference: 21/SC/0411; CPMS study ID: 54143.

## Consent

All participants involved provided informed consent prior to participation in the study.

## Conflicts of Interest

The authors declare no conflicts of interest.

## Supporting information


**Figure S1:** Methodological pathway used in this study.
**Figure S2:** Top metabolite variables driving variation in the Partial Least Squared‐Discriminant Analysis (PLS‐DA) comparing the urinary metabolomes from four study groups (*n* = 400); BC = Breast Cancer (*n* = 118), BBD = benign breast disease (*n* = 148), SC = symptom controls (*n* = 95), HC = Healthy Controls (*n* = 39). Suggested identifications are indicated linked to Variable Importance in Projection (VIP) score.
**Figure S3:** Supervised Partial Least Squares–Discriminant Analysis (PLS‐DA) of Urine Metabolomes Across Four Study Groups considered PLS‐DA of urine metabolomes for four study groups (*n* = 400); BC = Breast Cancer (*n* = 118), BBD = benign breast disease (*n* = 148), SC = symptom controls (*n* = 95), HC = Healthy Controls (*n* = 39) in six age categories (a) < 40, (b) 40–49, (c) 50–59, (d) 60–69, (e) 70–79 and (f) ≥ 80 years old.
**Figure S4:** Box and Whisker plots of the metabolites which significantly differ in urine samples from breast cancer patients from other groups. Supervised Partial Least Squares–Discriminant Analysis (PLS‐DA) of Urine Metabolomes Across Four Study Groups considered Metabolites showing significant increases or decreases in BC = Breast Cancer (*n* = 118) compared to BBD = benign breast disease (*n* = 148), SC = symptom controls (*n* = 95), HC = Healthy Controls (*n* = 39) groups; lactic acid/lactate (*p* = 0.00069068), lactose 6‐phosphate (*p* = 0.0012367), D‐ribose sugar (*p* = 0.00039174), dihydroxyindole (*p* = 0.0013661), hypoxanthine (*p* = 0.00089183), homovanillic acid (*p* = 0.00069068), glutamic acid (*p* = 0.00099174), L‐glutamic gamma‐semialdehyde (*p* = 0.00093306), histidine (*p* = 0.00054287), L‐valine (*p* = 0.0013564) and urea (*p* = 0.00069068). Comparisons used Analysis of variance (ANOVA) and false discovery rates [FDR correction (Benjamini–Hochberg)] which are given as the *p* values.

**Table S1:** Patient metadata.

**Table S2:** cam472018‐sup‐0003‐TableS2.xlsx. *m/z* matrix derived following metabolite fingerprinting.

**Table S3:** Differentially accumulated metabolites between breast cancer (BC), benign breast disease (BBD), symptom control (SC) and healthy control (HC) groups based on post hoc ANOVA.

**Table S4:** Summary of significant metabolites discriminating between breast cancer (BC) and healthy controls (HC) in urine, plasma and serum as identified by *t‐*test (corrected for false discovery rate).

## Data Availability

The raw data mentioned in the study are included in the Supporting Information. Further inquiries can be directed to the corresponding author.

## References

[1] S. Chen , Z. Cao , K. Prettner , et al., “Estimates and Projections of the Global Economic Cost of 29 Cancers in 204 Countries and Territories From 2020 to 2050,” JAMA Oncology 9, no. 4 (2023): 465–472. 36821107 10.1001/jamaoncol.2022.7826 PMC9951101

[2] M. Nigam and B. Nigam , “Triple Assessment of Breast—Gold Standard in Mass Screening for Breast Cancer Diagnosis,” IOSR Journal of Dental and Medical Sciences (IOSR‐JDMS) 7 (2013): 1–7.

[3] S. Niraula , N. Biswanger , P. Hu , P. Lambert , and K. Decker , “Incidence, Characteristics, and Outcomes of Interval Breast Cancers Compared With Screening‐Detected Breast Cancers,” JAMA Network Open 3, no. 9 (2020): e2018179. 32975573 10.1001/jamanetworkopen.2020.18179 PMC7519419

[4] N. Houssami , “Overdiagnosis of Breast Cancer in Population Screening: Does It Make Breast Screening Worthless?,” Cancer Biology & Medicine 14, no. 1 (2017): 1–8. 28443199 10.20892/j.issn.2095-3941.2016.0050 PMC5365181

[5] N. R. Payne , S. E. Hickman , R. Black , A. N. Priest , S. Hudson , and F. J. Gilbert , “Breast Density Effect on the Sensitivity of Digital Screening Mammography in a UK Cohort,” European Radiology 35, no. 1 (2025): 177–187. 39017933 10.1007/s00330-024-10951-w PMC11631811

[6] D. R. Schmidt , R. Patel , D. G. Kirsch , C. A. Lewis , M. G. Vander Heiden , and J. W. Locasale , “Metabolomics in Cancer Research and Emerging Applications in Clinical Oncology,” CA: A Cancer Journal for Clinicians 71, no. 4 (2021): 333–358. 33982817 10.3322/caac.21670 PMC8298088

[7] L. Yang , Y. Wang , H. Cai , S. Wang , Y. Shen , and C. Ke , “Application of Metabolomics in the Diagnosis of Breast Cancer: A Systematic Review,” Journal of Cancer 11, no. 9 (2020): 2540–2551. 32201524 10.7150/jca.37604 PMC7066003

[8] M. Yang , J. Jiang , L. Hua , et al., “Rapid Detection of Volatile Organic Metabolites in Urine by High‐Pressure Photoionization Mass Spectrometry for Breast Cancer Screening: A Pilot Study,” Metabolites 13, no. 7 (2023): 870. 37512577 10.3390/metabo13070870 PMC10385751

[9] J. Park , Y. Shin , T. Kim , D.‐H. Kim , and A. Lee , “Urinary Metabolites as Biomarkers for Diagnosis of Breast Cancer: A Preliminary Study,” Journal of Breast Disease 7 (2019): 44–51.

[10] D. T. Eniu , A. R. Chiorean , A. I. Socaciu , et al., “Blood and Urine Biomarkers in Invasive Ductal Breast Cancer: Mass Spectrometry Applied to Identify Metabolic Alterations,” Journal of Molecular Structure 1247 (2022): 131369.

[11] C. L. Silva , R. Perestrelo , F. Capelinha , H. Tomas , and J. S. Camara , “An Integrative Approach Based on GC‐qMS and NMR Metabolomics Data as a Comprehensive Strategy to Search Potential Breast Cancer Biomarkers,” Metabolomics 17, no. 8 (2021): 72. 34389918 10.1007/s11306-021-01823-1

[12] F. Zahran , R. Rashed , M. Omran , H. Darwish , and A. Belal , “Study on Urinary Candidate Metabolome for the Early Detection of Breast Cancer,” Indian Journal of Clinical Biochemistry 36, no. 3 (2021): 319–329. 34220007 10.1007/s12291-020-00905-6 PMC8215016

[13] L. Lin , Q. Yu , X. Yan , et al., “Direct Infusion Mass Spectrometry or Liquid Chromatography Mass Spectrometry for Human Metabonomics? A Serum Metabonomic Study of Kidney Cancer,” Analyst 135, no. 11 (2010): 2970–2978. 20856980 10.1039/c0an00265h

[14] R. González‐Domínguez , A. Sayago , and Á. Fernández‐Recamales , “Direct Infusion Mass Spectrometry for Metabolomic Phenotyping of Diseases,” Bioanalysis 9, no. 1 (2017): 131–148. 27921460 10.4155/bio-2016-0202

[15] J. Draper , A. J. Lloyd , R. Goodacre , and M. Beckmann , “Flow Infusion Electrospray Ionisation Mass Spectrometry for High Throughput, Non‐Targeted Metabolite Fingerprinting: A Review,” Metabolomics 9, no. Suppl 1 (2013): 4–29.

[16] R. Aguiar‐Ibáñez , Y. Mbous , S. Sharma , R. Chakali , and E. Chawla , “Barriers to Cancer Screening Uptake and Approaches to Overcome Them: A Systematic Literature Review,” Frontiers in Oncology 15 (2025): 1575820. 40842583 10.3389/fonc.2025.1575820 PMC12364675

[17] H. Zhu and B. E. Doğan , “American Joint Committee on Cancer's Staging System for Breast Cancer, Eighth Edition: Summary for Clinicians,” European Journal of Breast Health 17, no. 3 (2021): 234–238. 34263150 10.4274/ejbh.galenos.2021.2021-4-3 PMC8246053

[18] World Medical Association , “World Medical Association Declaration of Helsinki: Ethical Principles for Medical Research Involving Human Subjects,” JAMA 310, no. 20 (2013): 2191–2194. 24141714 10.1001/jama.2013.281053

[19] J. A. Kirwan , R. J. Weber , D. I. Broadhurst , and M. R. Viant , “Direct Infusion Mass Spectrometry Metabolomics Dataset: A Benchmark for Data Processing and Quality Control,” Scientific Data 1 (2014): 140012. 25977770 10.1038/sdata.2014.12 PMC4381748

[20] C. Marques , L. Liu , K. D. Duncan , and I. Lanekoff , “A Direct Infusion Probe for Rapid Metabolomics of Low‐Volume Samples,” Analytical Chemistry 94, no. 37 (2022): 12875–12883. 36070505 10.1021/acs.analchem.2c02918 PMC9494293

[21] J. P. Finch , T. Wilson , L. Lyons , H. Phillips , M. Beckmann , and J. Draper , “Spectral Binning as an Approach to Post‐Acquisition Processing of High Resolution FIE‐MS Metabolome Fingerprinting Data,” Metabolomics 18, no. 8 (2022): 64. 35917032 10.1007/s11306-022-01923-6 PMC9345815

[22] J. Draper , D. P. Enot , D. Parker , et al., “Metabolite Signal Identification in Accurate Mass Metabolomics Data With MZedDB, an Interactive *m/z* Annotation Tool Utilising Predicted Ionisation Behaviour ‘Rules’,” BMC Bioinformatics 10 (2009): 227. 19622150 10.1186/1471-2105-10-227 PMC2721842

[23] Z. Pang , Y. Lu , G. Zhou , et al., “MetaboAnalyst 6.0: Towards a Unified Platform for Metabolomics Data Processing, Analysis and Interpretation,” Nucleic Acids Research 52, no. W1 (2024): W398–W406. 38587201 10.1093/nar/gkae253 PMC11223798

[24] F. Rohart , B. Gautier , A. Singh , and K. A. Lê Cao , “mixOmics: An R Package for 'Omics Feature Selection and Multiple Data Integration,” PLoS Computational Biology 13, no. 11 (2017): e1005752. 29099853 10.1371/journal.pcbi.1005752 PMC5687754

[25] A. Singh , C. P. Shannon , B. Gautier , et al., “DIABLO: An Integrative Approach for Identifying Key Molecular Drivers From Multi‐Omics Assays,” Bioinformatics 35, no. 17 (2019): 3055–3062. 30657866 10.1093/bioinformatics/bty1054 PMC6735831

[26] K. Andrade de Oliveira , S. Sengupta , A. K. Yadav , and R. Clarke , “The Complex Nature of Heterogeneity and Its Roles in Breast Cancer Biology and Therapeutic Responsiveness,” Frontiers in Endocrinology 14 (2023): 1083048. 36909339 10.3389/fendo.2023.1083048 PMC9997040

[27] M. Hassan , L. Tutar , D. Sari‐Ak , A. Rasul , E. Basheer , and Y. Tutar , “Non‐Genetic Heterogeneity and Immune Subtyping in Breast Cancer: Implications for Immunotherapy and Targeted Therapeutics,” Translational Oncology 47 (2024): 102055. 39002207 10.1016/j.tranon.2024.102055 PMC11299575

[28] C. Blucher and S. C. Stadler , “Obesity and Breast Cancer: Current Insights on the Role of Fatty Acids and Lipid Metabolism in Promoting Breast Cancer Growth and Progression,” Frontiers in Endocrinology 8 (2017): 293. 29163362 10.3389/fendo.2017.00293 PMC5670108

[29] Y. J. Cha , E. S. Kim , and J. S. Koo , “Amino Acid Transporters and Glutamine Metabolism in Breast Cancer,” International Journal of Molecular Sciences 19, no. 3 (2018): 907. 29562706 10.3390/ijms19030907 PMC5877768

[30] E. Shin and J. S. Koo , “Glucose Metabolism and Glucose Transporters in Breast Cancer,” Frontiers in Cell and Development Biology 9 (2021): 728759. 10.3389/fcell.2021.728759 PMC8450384 34552932

[31] M. Tufail , C. H. Jiang , and N. Li , “Altered Metabolism in Cancer: Insights Into Energy Pathways and Therapeutic Targets,” Molecular Cancer 23, no. 1 (2024): 203. 39294640 10.1186/s12943-024-02119-3 PMC11409553

[32] S. Qiu , Y. Cai , H. Yao , et al., “Small Molecule Metabolites: Discovery of Biomarkers and Therapeutic Targets,” Signal Transduction and Targeted Therapy 8, no. 1 (2023): 132. 36941259 10.1038/s41392-023-01399-3 PMC10026263

[33] R. J. DeBerardinis and N. S. Chandel , “Fundamentals of Cancer Metabolism,” Science Advances 2, no. 5 (2016): e1600200. 27386546 10.1126/sciadv.1600200 PMC4928883

[34] C. M. Slupsky , H. Steed , T. H. Wells , et al., “Urine Metabolite Analysis Offers Potential Early Diagnosis of Ovarian and Breast Cancers,” Clinical Cancer Research 16, no. 23 (2010): 5835–5841. 20956617 10.1158/1078-0432.CCR-10-1434

[35] C. L. Silva , A. Olival , R. Perestrelo , P. Silva , H. Tomás , and J. S. Câmara , “Untargeted Urinary (1)H NMR‐Based Metabolomic Pattern as a Potential Platform in Breast Cancer Detection,” Metabolites 9, no. 11 (2019): 269. 31703396 10.3390/metabo9110269 PMC6918409

[36] C. L. Silva , M. Passos , and J. S. Câmara , “Solid Phase Microextraction, Mass Spectrometry and Metabolomic Approaches for Detection of Potential Urinary Cancer Biomarkers—A Powerful Strategy for Breast Cancer Diagnosis,” Talanta 89 (2012): 360–368. 22284503 10.1016/j.talanta.2011.12.041

[37] J. Beretov , V. C. Wasinger , E. K. Millar , P. Schwartz , P. H. Graham , and Y. Li , “Proteomic Analysis of Urine to Identify Breast Cancer Biomarker Candidates Using a Label‐Free LC‐MS/MS Approach,” PLoS One 10, no. 11 (2015): e0141876. 26544852 10.1371/journal.pone.0141876 PMC4636393

[38] M. Beckmann , D. Parker , D. P. Enot , E. Duval , and J. Draper , “High‐Throughput, Nontargeted Metabolite Fingerprinting Using Nominal Mass Flow Injection Electrospray Mass Spectrometry,” Nature Protocols 3, no. 3 (2008): 486–504. 18323818 10.1038/nprot.2007.500

[39] D. P. Overy , D. P. Enot , K. Tailliart , et al., “Explanatory Signal Interpretation and Metabolite Identification Strategies for Nominal Mass FIE‐MS Metabolite Fingerprints,” Nature Protocols 3, no. 3 (2008): 471–485. 18323817 10.1038/nprot.2007.512

[40] J. Shen , L. Yan , S. Liu , C. B. Ambrosone , and H. Zhao , “Plasma Metabolomic Profiles in Breast Cancer Patients and Healthy Controls: By Race and Tumor Receptor Subtypes,” Translational Oncology 6, no. 6 (2013): 757–765. 24466379 10.1593/tlo.13619 PMC3890711

[41] M. Jove , R. Collado , J. L. Quiles , et al., “A Plasma Metabolomic Signature Discloses Human Breast Cancer,” Oncotarget 8, no. 12 (2017): 19522–19533. 28076849 10.18632/oncotarget.14521 PMC5386702

[42] F. Kou , B. Zhu , W. Zhou , C. Lv , Y. Cheng , and H. Wei , “Targeted Metabolomics in the Cell Culture Media Reveals Increased Uptake of Branched Amino Acids by Breast Cancer Cells,” Analytical Biochemistry 624 (2021): 114192. 33812922 10.1016/j.ab.2021.114192

[43] Y. Liu , Y. Zhao , H. Song , et al., “Metabolic Reprogramming in Tumor Immune Microenvironment: Impact on Immune Cell Function and Therapeutic Implications,” Cancer Letters 597 (2024): 217076. 38906524 10.1016/j.canlet.2024.217076

[44] J. E. Bader , K. Voss , and J. C. Rathmell , “Targeting Metabolism to Improve the Tumor Microenvironment for Cancer Immunotherapy,” Molecular Cell 78, no. 6 (2020): 1019–1033. 32559423 10.1016/j.molcel.2020.05.034 PMC7339967

[45] G. Ma , Z. Zhang , P. Li , et al., “Reprogramming of Glutamine Metabolism and Its Impact on Immune Response in the Tumor Microenvironment,” Cell Communication and Signaling: CCS 20, no. 1 (2022): 114. 35897036 10.1186/s12964-022-00909-0 PMC9327201

[46] L. Fang , D. Gao , Z. Jiang , G. Li , and M. Li , “Glutamine's Double‐Edged Sword: Fueling Tumor Growth and Offering Therapeutic Hope,” Frontiers in Immunology 16 (2025): 1578940. 40276500 10.3389/fimmu.2025.1578940 PMC12018421

[47] H. Muranaka , R. Akinsola , S. Billet , et al., “Glutamine Supplementation as an Anticancer Strategy: A Potential Therapeutic Alternative to the Convention,” Cancers (Basel) 16, no. 5 (2024): 1057. 38473414 10.3390/cancers16051057 PMC10930819

[48] H. Nam , B. C. Chung , Y. Kim , K. Lee , and D. Lee , “Combining Tissue Transcriptomics and Urine Metabolomics for Breast Cancer Biomarker Identification,” Bioinformatics 25, no. 23 (2009): 3151–3157. 19783829 10.1093/bioinformatics/btp558

[49] D. E. Mahoney , J. B. Hiebert , A. Thimmesch , et al., “Understanding D‐Ribose and Mitochondrial Function,” Advances in Bioscience and Clinical Medicine 6, no. 1 (2018): 1–5. 29780691 10.7575/aiac.abcmed.v.6n.1p.1 PMC5959283

[50] J. Hong , G. Li , Q. Zhang , J. Ritter , W. Li , and P. L. Li , “D‐Ribose Induces Podocyte NLRP3 Inflammasome Activation and Glomerular Injury via AGEs/RAGE Pathway,” Frontiers in Cell and Developmental Biology 7 (2019): 259. 31737627 10.3389/fcell.2019.00259 PMC6831643

[51] Y. Yu , S. Liu , L. Yang , et al., “Roles of Reactive Oxygen Species in Inflammation and Cancer,” MedComm 5, no. 4 (2024): e519. 38576456 10.1002/mco2.519 PMC10993368

[52] D. Sun , X. Li , S. Nie , J. Liu , and S. Wang , “Disorders of Cancer Metabolism: The Therapeutic Potential of Cannabinoids,” Biomedicine & Pharmacotherapy 157 (2023): 113993. 36379120 10.1016/j.biopha.2022.113993

[53] X. Huang , B. Liu , and S. Shen , “Lipid Metabolism in Breast Cancer: From Basic Research to Clinical Application,” Cancers (Basel) 17, no. 4 (2025): 650. 40002245 10.3390/cancers17040650 PMC11852908

[54] H. Azimi , A. Jafari , M. Maralani , and H. Davoodi , “The Role of Histamine and Its Receptors in Breast Cancer: From Pathology to Therapeutic Targets,” Medical Oncology 41, no. 8 (2024): 190. 38951252 10.1007/s12032-024-02437-y

[55] M. Valko‐Rokytovska , P. Ocenas , A. Salayova , and Z. Kostecka , “Breast Cancer: Targeting of Steroid Hormones in Cancerogenesis and Diagnostics,” International Journal of Molecular Sciences 22, no. 11 (2021): 5878. 34070921 10.3390/ijms22115878 PMC8199112

[56] Y. Jang , “Bioactive Compounds Targeting Dihydroceramide and Their Therapeutic Potential in Cancer Treatment,” Cancers (Basel) 17, no. 5 (2025): 909. 40075756 10.3390/cancers17050909 PMC11898591

[57] P. A. Corsetto , S. Zava , A. M. Rizzo , and I. Colombo , “The Critical Impact of Sphingolipid Metabolism in Breast Cancer Progression and Drug Response,” International Journal of Molecular Sciences 24, no. 3 (2023): 2107. 36768427 10.3390/ijms24032107 PMC9916652

[58] A. Nounu , S. P. Kar , C. L. Relton , and R. C. Richmond , “Sex Steroid Hormones and Risk of Breast Cancer: A Two‐Sample Mendelian Randomization Study,” Breast Cancer Research 24, no. 1 (2022): 66. 36209141 10.1186/s13058-022-01553-9 PMC9548139

[59] R. Lopez‐Marure , E. Zapata‐Gomez , L. Rocha‐Zavaleta , et al., “Dehydroepiandrosterone Inhibits Events Related With the Metastatic Process in Breast Tumor Cell Lines,” Cancer Biology & Therapy 17, no. 9 (2016): 915–924. 27260851 10.1080/15384047.2016.1195047 PMC5036408

[60] H. Yuan , Y. Chen , Y. Hu , et al., “Disulfide Bond‐Driven Nanoassembly of Lipophilic Epirubicin Prodrugs for Breast Cancer Therapy,” Journal of Pharmaceutical Investigation 55 (2025): 889–902.

[61] M. Mathew , N. T. Nguyen , Y. D. Bhutia , S. Sivaprakasam , and V. Ganapathy , “Metabolic Signature of Warburg Effect in Cancer: An Effective and Obligatory Interplay Between Nutrient Transporters and Catabolic/Anabolic Pathways to Promote Tumor Growth,” Cancers (Basel) 16, no. 3 (2024): 504. 38339256 10.3390/cancers16030504 PMC10854907

[62] H. Suleiman , A. Emerson , P. M. Wilson , K. A. Mulligan , R. D. Ladner , and M. J. LaBonte , “Harnessing Nucleotide Metabolism and Immunity in Cancer: A Tumour Microenvironment Perspective,” FEBS Journal 292, no. 9 (2025): 2155–2172. 39308084 10.1111/febs.17278 PMC12062787

[63] E. S. Ali and I. Ben‐Sahra , “Regulation of Nucleotide Metabolism in Cancers and Immune Disorders,” Trends in Cell Biology 33, no. 11 (2023): 950–966. 36967301 10.1016/j.tcb.2023.03.003 PMC10518033

[64] H. C. Yoo , Y. C. Yu , Y. Sung , and J. M. Han , “Glutamine Reliance in Cell Metabolism,” Experimental & Molecular Medicine 52, no. 9 (2020): 1496–1516. 32943735 10.1038/s12276-020-00504-8 PMC8080614

[65] I. T. Helenius , H. R. Madala , and J. J. Yeh , “An Asp to Strike Out Cancer? Therapeutic Possibilities Arising From Aspartate's Emerging Roles in Cell Proliferation and Survival,” Biomolecules 11, no. 11 (2021): 1666. 34827664 10.3390/biom11111666 PMC8615858

[66] A. Kalezic , M. Udicki , B. Srdic Galic , et al., “Tissue‐Specific Warburg Effect in Breast Cancer and Cancer‐Associated Adipose Tissue‐Relationship Between AMPK and Glycolysis,” Cancers (Basel) 13, no. 11 (2021): 2731. 34073074 10.3390/cancers13112731 PMC8198826

[67] G. M. Rather , A. A. Pramono , Z. Szekely , J. R. Bertino , and P. M. Tedeschi , “In Cancer, All Roads Lead to NADPH,” Pharmacology & Therapeutics 226 (2021): 107864. 33894275 10.1016/j.pharmthera.2021.107864

[68] X. Shen , S. Miao , Y. Zhang , et al., “Stearic Acid Metabolism in Human Health and Disease,” Clinical Nutrition 44 (2025): 222–238. 39709650 10.1016/j.clnu.2024.12.012

[69] V. Panwar , A. Singh , M. Bhatt , et al., “Multifaceted Role of mTOR (Mammalian Target of Rapamycin) Signaling Pathway in Human Health and Disease,” Signal Transduction and Targeted Therapy 8, no. 1 (2023): 375. 37779156 10.1038/s41392-023-01608-z PMC10543444

[70] M. S. Hussain , A. S. Altamimi , M. Afzal , et al., “From Carcinogenesis to Therapeutic Avenues: lncRNAs and mTOR Crosstalk in Lung Cancer,” Pathology, Research and Practice 253 (2024): 155015. 38103364 10.1016/j.prp.2023.155015

[71] J. R. Almeida , L. B. Silva , I. Bos , P. J. Visser , and J. L. Oliveira , “A Methodology for Cohort Harmonisation in Multicentre Clinical Research,” Informatics in Medicine Unlocked 27 (2021): 100760, 10.1016/j.imu.2021.100760.

[72] K. W. A. Tang , Q. C. Toh , and B. W. Teo , “Normalisation of Urinary Biomarkers to Creatinine for Clinical Practice and Research—When and Why,” Singapore Medical Journal 56, no. 1 (2015): 7–10, 10.11622/smedj.2015003. 25640093 PMC4325562

