# Supplementary figures and images for: Metabolic Fingerprinting of Urine Reveals Metabolite Changes in Women With Breast Cancer
Source: Cancer Med. 2026 Jun 10;15(6):e72018. doi: 10.1002/cam4.72018 (PMC13253611; doi:10.1002/cam4.72018)

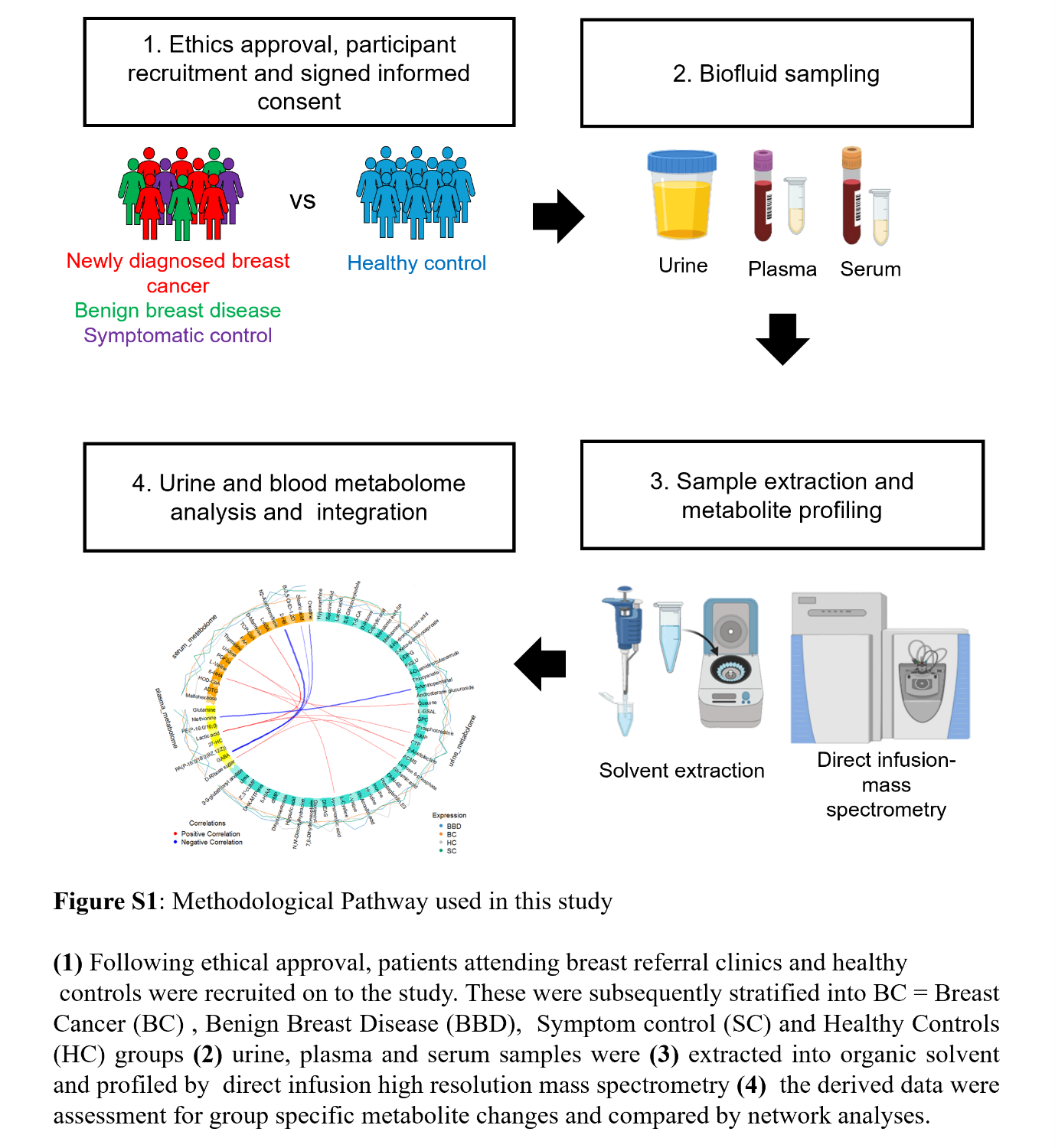


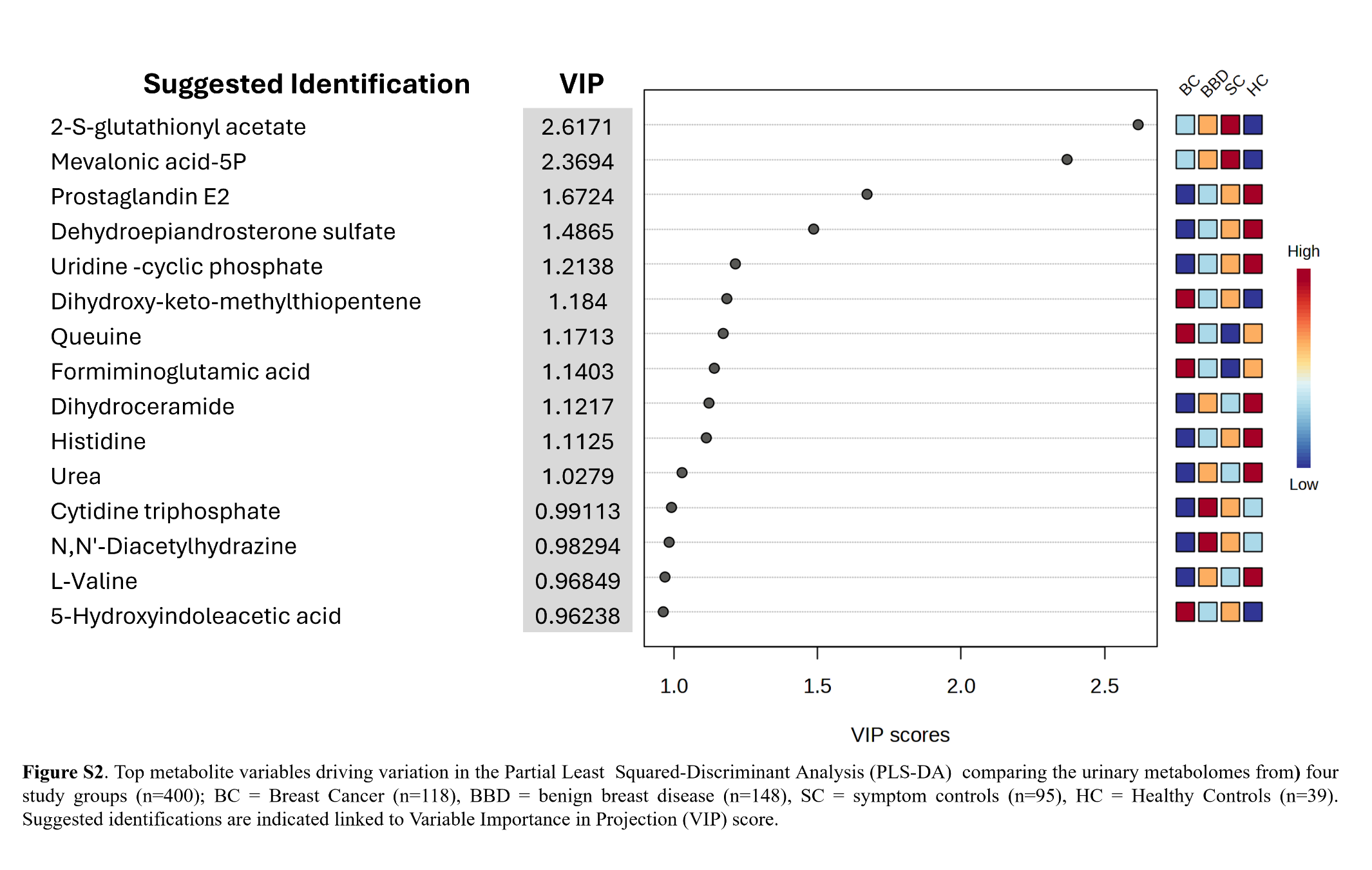


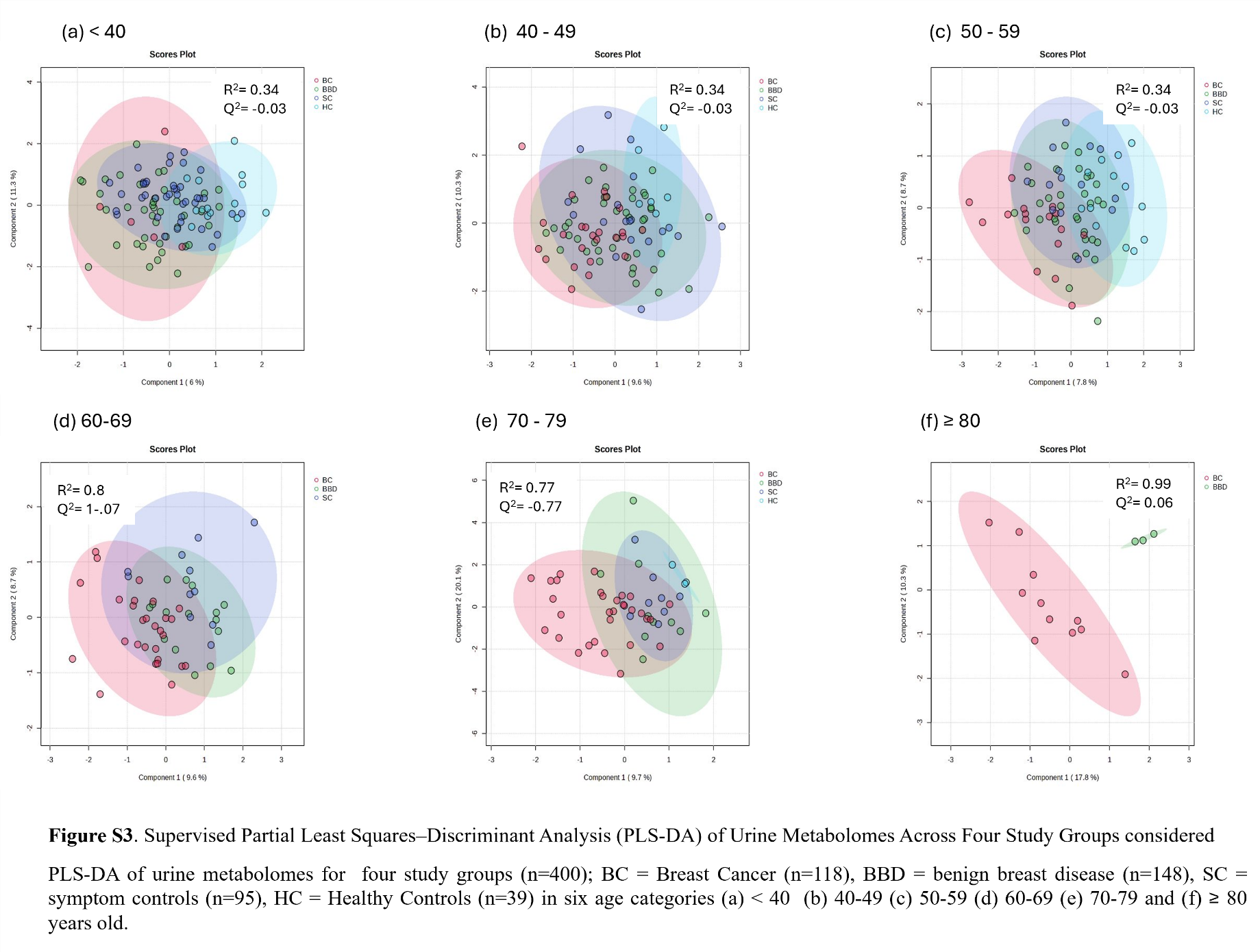


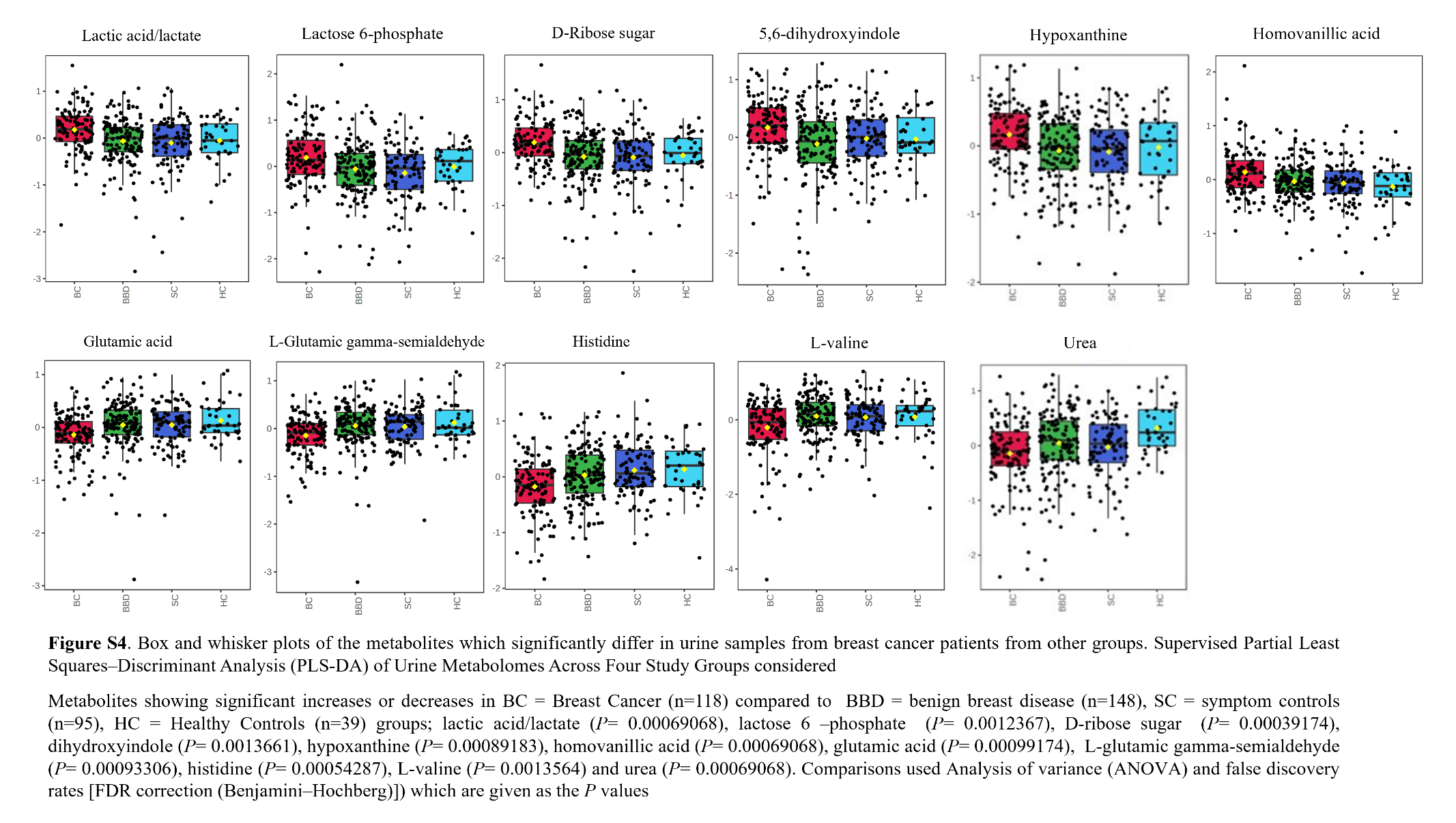

Supplement: Supplementary file 1 — Figure S1: Methodological pathway used in this study. Figure S2: Top metabolite variables driving variation in the Partial Least Squared‐Discriminant Analysis (PLS‐DA) comparing the urinary metabolomes from four study groups (n = 400); BC = Breast Cancer (n = 118), BBD = benign breast disease (n = 148), SC = symptom controls (n = 95), HC = Healthy Controls (n = 39). Suggested identifications are indicated linked to Variable Importance in Projection (VIP) score. Figure S3: Supervised Partial Least Squares–Discriminant Analysis (PLS‐DA) of Urine Metabolomes Across Four Study Groups considered PLS‐DA of urine metabolomes for four study groups (n = 400); BC = Breast Cancer (n = 118), BBD = benign breast disease (n = 148), SC = symptom controls (n = 95), HC = Healthy Controls (n = 39) in six age categories (a) < 40, (b) 40–49, (c) 50–59, (d) 60–69, (e) 70–79 and (f) ≥ 80 years old. Figure S4: Box and Whisker plots of the metabolites which significantly differ in urine samples from breast cancer patients from other groups. Supervised Partial Least Squares–Discriminant Analysis (PLS‐DA) of Urine Metabolomes Across Four Study Groups considered Metabolites showing significant increases or decreases in BC = Breast Cancer (n = 118) compared to BBD = benign breast disease (n = 148), SC = symptom controls (n = 95), HC = Healthy Controls (n = 39) groups; lactic acid/lactate (p = 0.00069068), lactose 6‐phosphate (p = 0.0012367), D‐ribose sugar (p = 0.00039174), dihydroxyindole (p = 0.0013661), hypoxanthine (p = 0.00089183), homovanillic acid (p = 0.00069068), glutamic acid (p = 0.00099174), L‐glutamic gamma‐semialdehyde (p = 0.00093306), histidine (p = 0.00054287), L‐valine (p = 0.0013564) and urea (p = 0.00069068). Comparisons used Analysis of variance (ANOVA) and false discovery rates [FDR correction (Benjamini–Hochberg)] which are given as the p values. [file CAM4-15-e72018-s001.docx]
