# Supplementary material for: Metabolic Fingerprinting of Urine Reveals Metabolite Changes in Women With Breast Cancer
Source: Cancer Med. 2026 Jun 10;15(6):e72018. doi: 10.1002/cam4.72018 (PMC13253611; doi:10.1002/cam4.72018)
Supplement: Supplementary file 4 — Table S3: Differentially accumulated metabolites between breast cancer (BC), benign breast disease (BBD), symptom control (SC) and healthy control (HC) groups based on post hoc ANOVA. [file CAM4-15-e72018-s005.docx]

**Table S3.** Differentially expressed metabolites between breast cancer (BC), benign breast disease (BBD), symptom control (SC) and healthy control (HC) groups based on *post-hoc* ANOVA.

| Metabolite | t.stat | p.value | log10(p) | FDR |
| --- | --- | --- | --- | --- |
| 1,2-Dihydroxy-3-keto-5-methylthiopentene | 5.9025 | 5.03 x 10-08 | 7.2981 | 1.12 x 10-05 |
| Glutamine | 5.4318 | 4.00 x 10-07 | 6.3984 | 4.46 x 10-05 |
| Dihydroceramide | -4.879 | 4.08 x 10-06 | 5.3892 | 0.000202 |
| Palmitoylcarnitine | -4.8657 | 4.31 x 10-06 | 5.3657 | 0.000202 |
| Thioguanine | 4.8532 | 4.53 x 10-06 | 5.3436 | 0.000202 |
| 2-Keto-6-aminocaproate | 4.7675 | 6.42 x 10-06 | 5.1928 | 0.000212 |
| N4-Acetylaminobutanal | 4.7579 | 6.67 x 10-06 | 5.1759 | 0.000212 |
| 1,3,7-Trimethyluric acid | 4.6546 | 1.01 x 10-05 | 4.9963 | 0.000248 |
| Gluconic acid | 4.6441 | 1.05 x 10-05 | 4.9782 | 0.000248 |
| Glucosamine 6-phosphate | 4.6302 | 1.11 x 10-05 | 4.9542 | 0.000248 |
| Homovanillic acid | 4.5913 | 1.30 x 10-05 | 4.8872 | 0.000259 |
| 3-Mercaptolactic acid | 4.5565 | 1.49 x 10-05 | 4.8278 | 0.000259 |
| Oxoadipic acid | 4.5513 | 1.52 x 10-05 | 4.8189 | 0.000259 |
| 1-Pyrroline-5-carboxylic acid | 4.5333 | 1.63 x 10-05 | 4.7882 | 0.000259 |
| Anandamide | -4.4233 | 2.50 x 10-05 | 4.6023 | 0.000371 |
| N-Acetyl-L-glutamate 5-semialdehyde | 4.3926 | 2.81 x 10-05 | 4.5508 | 0.000392 |
| S-Hydroxymethylglutathione | 4.3467 | 3.35 x 10-05 | 4.4745 | 0.00044 |
| L-Arabitol | 4.3201 | 3.71 x 10-05 | 4.4304 | 0.00046 |
| Ketoleucine | 4.2008 | 5.83 x 10-05 | 4.2345 | 0.000684 |
| L-Fucose | 4.14 | 7.31 x 10-05 | 4.1361 | 0.000815 |
| 5-Hydroxyindoleacetic acid | 4.1169 | 7.96 x 10-05 | 4.0989 | 0.000846 |
| Methionine | -4.0884 | 8.85 x 10-05 | 4.0533 | 0.000897 |
| 13-HODE | -4.0464 | 0.000103 | 3.9863 | 0.00091 |
| (R)-5-Diphosphomevalonic acid | 4.0354 | 0.000107 | 3.9688 | 0.00091 |
| Tetrahydrocorticosterone | -4.021 | 0.000113 | 3.9459 | 0.00091 |
| O-Phosphoethanolamine | -4.0208 | 0.000113 | 3.9457 | 0.00091 |
| MG(0:0/20:4(5Z,8Z,11Z,14Z)/0:0) | -4.0187 | 0.000114 | 3.9424 | 0.00091 |
| LysoPA(P-16:0/0:0) | -4.0185 | 0.000114 | 3.9419 | 0.00091 |
| Ubiquinol 8 | -3.9876 | 0.000128 | 3.8932 | 0.000983 |
| Glycerophosphocholine | -3.9714 | 0.000136 | 3.8678 | 0.001001 |
| 17-Hydroxyprogesterone | -3.9644 | 0.000139 | 3.8567 | 0.001001 |
| Uridine | -3.9133 | 0.000167 | 3.7769 | 0.001138 |
| 7alpha-Hydroxy-3-oxo-4-cholestenoate | -3.9114 | 0.000168 | 3.7738 | 0.001138 |
| Biliverdin | -3.9009 | 0.000175 | 3.7575 | 0.001146 |
| Cholic acid | -3.8758 | 0.000191 | 3.7186 | 0.001163 |
| Progesterone | -3.873 | 0.000193 | 3.7143 | 0.001163 |
| 15-Keto-prostaglandin x 102 | -3.8627 | 0.0002 | 3.6984 | 0.001163 |
| Succinic acid | 3.8585 | 0.000203 | 3.6919 | 0.001163 |
| D-Ribose | 3.8583 | 0.000203 | 3.6916 | 0.001163 |
| 3-Butynoate | 3.8504 | 0.000209 | 3.6795 | 0.001166 |
| D-Pantothenoyl-L-cysteine | -3.8358 | 0.00022 | 3.6569 | 0.001198 |
| dIMP | 3.8215 | 0.000232 | 3.635 | 0.001231 |
| 6-Hydroxyhexanoic acid | 3.7999 | 0.00025 | 3.6019 | 0.001297 |
| Choline | -3.7899 | 0.000259 | 3.5866 | 0.001309 |
| 2-Methoxy-estradiol-17b 3-glucuronide | -3.78 | 0.000268 | 3.5715 | 0.001309 |
| 17-beta- x 10stradiol-3-glucuronide | -3.7781 | 0.00027 | 3.5686 | 0.001309 |
| Monodehydroascorbate | 3.7702 | 0.000278 | 3.5566 | 0.001317 |
| Threonic acid | 3.7578 | 0.00029 | 3.5378 | 0.001341 |
| 5-Methoxyindoleacetate | 3.7531 | 0.000295 | 3.5306 | 0.001341 |
| Urobilinogen | -3.7366 | 0.000312 | 3.5056 | 0.001392 |
| Androsterone glucuronide | -3.7227 | 0.000328 | 3.4847 | 0.001426 |
| D-Glucose | 3.715 | 0.000336 | 3.4732 | 0.001426 |
| x 10strone glucuronide | -3.7057 | 0.000347 | 3.4591 | 0.001426 |
| Sepiapterin | 3.6988 | 0.000356 | 3.4488 | 0.001426 |
| Aminoadipic acid | 3.6963 | 0.000359 | 3.445 | 0.001426 |
| Cortisone | -3.6947 | 0.000361 | 3.4425 | 0.001426 |
| Homovanillin | -3.6877 | 0.00037 | 3.4321 | 0.001426 |
| Leukotriene A4 | -3.6847 | 0.000374 | 3.4277 | 0.001426 |
| Prostaglandin G2 | -3.6762 | 0.000385 | 3.4149 | 0.001426 |
| Prostaglandin x 102 | -3.6733 | 0.000389 | 3.4105 | 0.001426 |
| Proline | -3.6647 | 0.0004 | 3.3977 | 0.001426 |
| Thymidine | 3.6647 | 0.0004 | 3.3977 | 0.001426 |
| alpha-Ketoisovaleric acid | 3.6588 | 0.000408 | 3.389 | 0.001426 |
| Homogentisic acid | 3.6581 | 0.000409 | 3.3879 | 0.001426 |
| 4-Coumaryl alcohol | 3.6384 | 0.000438 | 3.3586 | 0.001503 |
| Serine | 3.6265 | 0.000456 | 3.3409 | 0.001522 |
| cis-Aconitic acid | 3.6258 | 0.000457 | 3.3398 | 0.001522 |
| Ubiquinone-1 | -3.6203 | 0.000466 | 3.3317 | 0.001528 |
| Dihydropteridine | 3.6025 | 0.000495 | 3.3054 | 0.001543 |
| 5-Acetylamino-6-formylamino-3-methyluracil | 3.6015 | 0.000497 | 3.3039 | 0.001543 |
| 11-Dehydrocorticosterone | -3.5939 | 0.00051 | 3.2927 | 0.001543 |
| Urocanic acid | 3.593 | 0.000511 | 3.2914 | 0.001543 |
| 2-Methoxyestrone | -3.5927 | 0.000512 | 3.2909 | 0.001543 |
| 3-Hydroxylidocaine | -3.5927 | 0.000512 | 3.2908 | 0.001543 |
| x 10striol | -3.5874 | 0.000521 | 3.2831 | 0.001549 |
| Adenosylcobalamin | -3.5782 | 0.000538 | 3.2695 | 0.001558 |
| Quinine | -3.5779 | 0.000538 | 3.2692 | 0.001558 |
| Thyrotropin releasing hormone | -3.568 | 0.000556 | 3.2546 | 0.001591 |
| N2-Acetylornithine | 3.5485 | 0.000594 | 3.226 | 0.001677 |
| Lactic acid | 3.5282 | 0.000636 | 3.1964 | 0.001774 |
| Azelaic acid | -3.5138 | 0.000668 | 3.1755 | 0.001818 |
| 5-Hydroxy-L-tryptophan | 3.5133 | 0.000669 | 3.1748 | 0.001818 |
| Thromboxane A2 | -3.5048 | 0.000688 | 3.1624 | 0.001849 |
| Caprylic acid | 3.4897 | 0.000723 | 3.1406 | 0.001911 |
| 5-Hydroxyeicosatetraenoic acid | -3.4877 | 0.000728 | 3.1377 | 0.001911 |
| N-Trimethyl-2-aminoethylphosphonate | -3.4777 | 0.000753 | 3.1233 | 0.001912 |
| Bilirubin | -3.4769 | 0.000755 | 3.1222 | 0.001912 |
| Inosinic acid | 3.4755 | 0.000759 | 3.12 | 0.001912 |
| Pelargonic acid | 3.4736 | 0.000763 | 3.1174 | 0.001912 |
| gamma-Glutamylcysteine | -3.4636 | 0.000789 | 3.103 | 0.001938 |
| 13-OxoOD x 10 | -3.4608 | 0.000796 | 3.099 | 0.001938 |
| L-Glutamic gamma-semialdehyde | -3.4595 | 0.0008 | 3.0971 | 0.001938 |
| 5,10-Methenyltetrahydrofolic acid | -3.43 | 0.000882 | 3.0547 | 0.00208 |
| Acetic acid | 3.4292 | 0.000884 | 3.0536 | 0.00208 |
| L-Cysteine | 3.4284 | 0.000886 | 3.0525 | 0.00208 |
| dUMP | 3.4133 | 0.000931 | 3.0309 | 0.002147 |
| Norepinephrine | 3.4124 | 0.000934 | 3.0297 | 0.002147 |
| 10-Formyltetrahydrofolate | -3.3986 | 0.000977 | 3.01 | 0.00221 |
| Deoxyuridine | 3.3974 | 0.000981 | 3.0084 | 0.00221 |
| Coprocholic acid | -3.3928 | 0.000996 | 3.0018 | 0.002221 |
| x 10rythritol | 3.3792 | 0.001041 | 2.9825 | 0.002299 |
| Porphobilinogen | 3.3735 | 0.001061 | 2.9744 | 0.002306 |
| Glutamic acid | 3.3722 | 0.001065 | 2.9726 | 0.002306 |
| Aminoacetone | 3.3418 | 0.001175 | 2.9298 | 0.002504 |
| NPC | -3.3408 | 0.001179 | 2.9285 | 0.002504 |
| Sphinganine 1-phosphate | -3.3375 | 0.001192 | 2.9237 | 0.002508 |
| 7a-Hydroxydehydroepiandrosterone | -3.3208 | 0.001258 | 2.9004 | 0.002617 |
| Prostaglandin x 101 | -3.3184 | 0.001267 | 2.8971 | 0.002617 |
| 3-Hydroxyanthranilic acid | 3.3075 | 0.001313 | 2.8817 | 0.002686 |
| Deoxycholic acid | -3.2997 | 0.001346 | 2.871 | 0.002729 |
| Androstenedione | -3.2925 | 0.001378 | 2.8609 | 0.002768 |
| Chitobiose | -3.2598 | 0.001529 | 2.8157 | 0.003044 |
| 13-L-Hydroperoxylinoleic acid | -3.2548 | 0.001554 | 2.8087 | 0.003066 |
| Mevalonic acid-5P | 3.2348 | 0.001655 | 2.7811 | 0.003217 |
| L-Carnitine | 3.2283 | 0.00169 | 2.7722 | 0.003217 |
| Carbamazepine | 3.2275 | 0.001694 | 2.7711 | 0.003217 |
| 3,4-Dihydroxymandelic acid | 3.2271 | 0.001696 | 2.7706 | 0.003217 |
| 4-Trimethylammoniobutanal | -3.2243 | 0.001711 | 2.7668 | 0.003217 |
| trans-1,2-Dihydrobenzene-1,2-diol | 3.2227 | 0.00172 | 2.7645 | 0.003217 |
| Melibiitol | -3.2206 | 0.001731 | 2.7617 | 0.003217 |
| 5,6-DH x 10T | -3.2142 | 0.001767 | 2.7529 | 0.003256 |
| Pyridoxamine | 3.2116 | 0.001781 | 2.7493 | 0.003256 |
| S-(2-Methylbutanoyl)-dihydrolipoamide | -3.206 | 0.001812 | 2.7418 | 0.003286 |
| x 10laidic acid | -3.2008 | 0.001842 | 2.7346 | 0.003313 |
| 3-Hydroxykynurenamine | 3.1949 | 0.001877 | 2.7266 | 0.003348 |
| Perillic acid | -3.1764 | 0.001989 | 2.7014 | 0.00352 |
| 2- x 10thylidene-1,5-dimethyl-3,3-diphenylpyrrolidine | -3.1721 | 0.002016 | 2.6956 | 0.003539 |
| Leucine | 3.1576 | 0.002109 | 2.6759 | 0.003674 |
| Dehydroepiandrosterone sulfate | -3.1544 | 0.00213 | 2.6717 | 0.003682 |
| N-Acetyl-L-glutamic acid | 3.1413 | 0.002218 | 2.654 | 0.003792 |
| Lithocholic acid | -3.1381 | 0.002241 | 2.6496 | 0.003792 |
| D-4'-Phosphopantothenate | -3.1375 | 0.002245 | 2.6488 | 0.003792 |
| 3-Methoxy-4-hydroxyphenylglycol glucuronide | 3.1298 | 0.002299 | 2.6385 | 0.003835 |
| S-Formylglutathione | 3.1269 | 0.00232 | 2.6345 | 0.003835 |
| N1-Acetylspermine | -3.1266 | 0.002322 | 2.6342 | 0.003835 |
| Phenylacetic acid | -3.1205 | 0.002366 | 2.626 | 0.003877 |
| 10Z-Nonadecenoic acid | -3.1183 | 0.002382 | 2.6231 | 0.003877 |
| Citric acid | 3.115 | 0.002407 | 2.6186 | 0.003889 |
| S-Acetyldihydrolipoamide | 3.1061 | 0.002473 | 2.6068 | 0.003955 |
| (S)-Propane-1,2-diol | 3.1049 | 0.002483 | 2.6051 | 0.003955 |
| Sulfite | 3.0842 | 0.002645 | 2.5775 | 0.004157 |
| alpha-Linolenic acid | -3.0827 | 0.002658 | 2.5755 | 0.004157 |
| Itaconic acid | 3.0817 | 0.002666 | 2.5742 | 0.004157 |
| Dolichyl diphosphate | 3.0674 | 0.002785 | 2.5552 | 0.004283 |
| 2,3-Butanediol | 3.0674 | 0.002785 | 2.5552 | 0.004283 |
| Xanthosine | 3.0618 | 0.002833 | 2.5478 | 0.004327 |
| 3a,7b,12a-Trihydroxy-5a-Cholanoic acid | -3.0373 | 0.003052 | 2.5155 | 0.004629 |
| Dodecanoic acid | -3.0325 | 0.003096 | 2.5092 | 0.004665 |
| Propynoic acid | 3.024 | 0.003177 | 2.498 | 0.004755 |
| Docosa-4,7,10,13,16-pentaenoyl CoA | -3.0047 | 0.003367 | 2.4727 | 0.004998 |
| Phenylacetaldehyde | -3.0023 | 0.003392 | 2.4696 | 0.004998 |
| L-2,3-Dihydrodipicolinate | 3.0009 | 0.003407 | 2.4677 | 0.004998 |
| Dihydrolipoate | 2.9817 | 0.003608 | 2.4427 | 0.005259 |
| 5a-Pregnane-3,20-dione | -2.9776 | 0.003652 | 2.4374 | 0.005289 |
| 5-Fluorouridine monophosphate | -2.9722 | 0.003712 | 2.4304 | 0.00534 |
| Butyric acid | 2.9672 | 0.003768 | 2.4239 | 0.005386 |
| 4-Hydroxybenzoic acid | -2.96 | 0.00385 | 2.4146 | 0.005403 |
| 3-Butyn-1-al | 2.9599 | 0.003851 | 2.4145 | 0.005403 |
| Quinolinic acid | 2.9597 | 0.003854 | 2.4141 | 0.005403 |
| Urea | -2.9577 | 0.003877 | 2.4115 | 0.005403 |
| Hydroquinone | 2.9482 | 0.003987 | 2.3994 | 0.005522 |
| 6-(alpha-D-Glucosaminyl)-1D-myo-inositol | 2.9455 | 0.004019 | 2.3959 | 0.005533 |
| Benzo[a]pyrene-4,5-oxide | -2.9415 | 0.004068 | 2.3906 | 0.00555 |
| Ornithine | 2.9403 | 0.004082 | 2.3891 | 0.00555 |
| 3beta-hydroxy-4beta-methyl-5alpha-cholest-7-ene-4alpha-carbaldehyde | -2.9362 | 0.004132 | 2.3838 | 0.005584 |
| 2-Methoxyestrone 3-glucuronide | -2.9323 | 0.004179 | 2.3789 | 0.005614 |
| Arachidonic acid | -2.911 | 0.004451 | 2.3515 | 0.005944 |
| x 10stradiol | -2.9084 | 0.004486 | 2.3482 | 0.005954 |
| N-Acetylmuramoyl-Ala | -2.8993 | 0.004607 | 2.3366 | 0.006068 |
| 5-Fluorouridine | 2.8979 | 0.004626 | 2.3348 | 0.006068 |
| Diketogulonic acid | -2.895 | 0.004666 | 2.3311 | 0.006077 |
| 1-Naphthaldehyde | -2.8934 | 0.004688 | 2.3291 | 0.006077 |
| Phenylpyruvic acid | 2.8691 | 0.005033 | 2.2982 | 0.006487 |
| Thiodiacetic acid | 2.8656 | 0.005084 | 2.2938 | 0.006509 |
| Dihomo-gamma-linolenic acid | -2.864 | 0.005108 | 2.2918 | 0.006509 |
| Argininosuccinic acid | -2.8596 | 0.005174 | 2.2862 | 0.006555 |
| Creatinine | 2.8508 | 0.005307 | 2.2751 | 0.006651 |
| Thiamine monophosphate | 2.8507 | 0.005309 | 2.275 | 0.006651 |
| Uric acid | -2.8443 | 0.005408 | 2.267 | 0.006718 |
| 3-Hydroxyquinine | 2.8428 | 0.005432 | 2.265 | 0.006718 |
| Taurine | -2.839 | 0.005492 | 2.2602 | 0.006718 |
| Pantetheine | -2.8383 | 0.005503 | 2.2594 | 0.006718 |
| Nebularine | 2.8377 | 0.005513 | 2.2586 | 0.006718 |
| Acetyl-N-formyl-5-methoxykynurenamine | 2.8301 | 0.005635 | 2.2491 | 0.00683 |
| 7,8-Dihydropteroic acid | -2.8225 | 0.005761 | 2.2395 | 0.006944 |
| Dihydrothymine | 2.8157 | 0.005874 | 2.231 | 0.007043 |
| Inosine | -2.81 | 0.005971 | 2.224 | 0.00712 |
| 3'-Ketolactose | 2.8076 | 0.006012 | 2.221 | 0.007131 |
| Benzo[a]pyrene-9,10-oxide | 2.7991 | 0.006162 | 2.2103 | 0.007239 |
| 20-Hydroxy-leukotriene x 104 | -2.7982 | 0.006177 | 2.2093 | 0.007239 |
| Alpha-Lactose | 2.7969 | 0.0062 | 2.2076 | 0.007239 |
| (1R)-Hydroxy-(2R)-N-acetyl-L-cysteinyl-1,2-dihydronaphthalene | 2.793 | 0.00627 | 2.2027 | 0.007283 |
| Tetrahydrocortisol | -2.7909 | 0.006308 | 2.2001 | 0.007289 |
| 4-Nitrophenol | 2.7867 | 0.006384 | 2.1949 | 0.007339 |
| S-Adenosylmethionine | -2.7836 | 0.00644 | 2.1911 | 0.007364 |
| Picolinic acid | 2.7794 | 0.006519 | 2.1859 | 0.007387 |
| N-Didesmethyl-tamoxifen | -2.7783 | 0.006539 | 2.1845 | 0.007387 |
| Stearic acid | -2.7755 | 0.006591 | 2.181 | 0.007387 |
| Histidine | 2.7754 | 0.006592 | 2.181 | 0.007387 |
| Myristic acid | -2.771 | 0.006676 | 2.1755 | 0.007443 |
| 17alpha,21-Dihydroxypregnenolone | -2.7672 | 0.006749 | 2.1708 | 0.007488 |
| Phenylalanine | 2.7651 | 0.006788 | 2.1682 | 0.007494 |
| Pyruvic acid | 2.7611 | 0.006866 | 2.1633 | 0.007519 |
| 13(S)-Hydroperoxylinolenic acid | -2.7605 | 0.006878 | 2.1625 | 0.007519 |
| Glutathione episulfonium ion | -2.7513 | 0.00706 | 2.1512 | 0.00768 |
| 9(S)-HPOD x 10 | -2.7377 | 0.007337 | 2.1345 | 0.007943 |
| Heme O | 2.7313 | 0.00747 | 2.1267 | 0.008043 |
| 4-Hydroxybenzaldehyde | -2.7297 | 0.007504 | 2.1247 | 0.008043 |
| 2'-Deoxyguanosine 5'-monophosphate | 2.7281 | 0.007538 | 2.1227 | 0.008043 |
| S-Adenosylhomocysteine | 2.7259 | 0.007584 | 2.1201 | 0.008052 |
| 5-Hydroxyindoleacetaldehyde | -2.7243 | 0.007619 | 2.1181 | 0.008052 |
| Palmitic acid | -2.7179 | 0.007756 | 2.1103 | 0.008159 |
| Cortisol | -2.7116 | 0.007896 | 2.1026 | 0.008266 |
| Prostaglandin x 103 | -2.7096 | 0.00794 | 2.1002 | 0.008274 |
| Aspartylglycosamine | 2.7052 | 0.008038 | 2.0948 | 0.008288 |
| L-Tyrosine | -2.7044 | 0.008056 | 2.0939 | 0.008288 |
| Lithocholyltaurine | 2.704 | 0.008065 | 2.0934 | 0.008288 |
| N1-Acetylspermidine | 2.6986 | 0.008188 | 2.0868 | 0.008376 |
| cis-Melilotoside | -2.6894 | 0.008402 | 2.0756 | 0.008555 |
| Arachidic acid | -2.6743 | 0.008761 | 2.0574 | 0.008857 |
| Testosterone glucuronide | -2.673 | 0.008794 | 2.0558 | 0.008857 |
| 4-Imidazolone-5-propionic acid | 2.672 | 0.008817 | 2.0547 | 0.008857 |
