# Supplementary material for: Metabolic Fingerprinting of Urine Reveals Metabolite Changes in Women With Breast Cancer
Source: Cancer Med. 2026 Jun 10;15(6):e72018. doi: 10.1002/cam4.72018 (PMC13253611; doi:10.1002/cam4.72018)
Supplement: Supplementary file 5 — Table S4: Summary of significant metabolites discriminating between breast cancer (BC) and healthy controls (HC) in urine, plasma and serum as identified by t‐test (corrected for false discovery rate). [file CAM4-15-e72018-s002.docx]

**Table S4.** Summary of significant metabolites discriminating between breast cancer (BC) and healthy controls (HC) in urine, plasma and serum as identified by *t-*test (corrected for false discovery rate).

| K x 10GG ID | Metabolite | p-value | | |
| --- | --- | --- | --- | --- |
|  |  | Urine | Plasma | Serum |
| C16172 | (6Z,9Z,12Z,15Z,18Z)-Tetracosapentaenoyl-CoA | NA | 0.00042738 | NA |
| C16196 | 1,2-Dihydroxynaphthalene-6-sulfonate | 0.00034136 | NA | NA |
| ^b^C00174 | 10Z-Nonadecenoic acid | NA | 1.39 x 10-05 | 0.00055818 |
| C04717 | 13-L-Hydroperoxylinoleic acid | NA | 0.00014066 | NA |
| C05487 | 17alpha,21-Dihydroxypregnenolone | NA | NA | 1.89 x 10-07 |
| C04932 | 2,3,2',3'-Tetrakis(3-hydroxytetradecanoyl)-D-glucosaminyl-1,6-beta-D-glucosamine 1-phosphate | NA | 0.0020313 | NA |
| C04409 | 2-Amino-3-carboxymuconic acid semialdehyde | 0.0017219 | NA | NA |
| ^a^C03239 | 2-Keto-6-aminocaproate | 0.0013997 | 0.0072165 | NA |
| C11131 | 2-Methoxy-estradiol-17b 3-glucuronide | NA | 1.35 x 10-06 | NA |
| C05450 | 3a,7a,12a,24-Tetrahydroxy-5b-cholestanoyl-CoA | NA | 0.0022239 | NA |
| C05467 | 3a,7a,12a-Trihydroxy-5b-24-oxocholestanoyl-CoA | NA | 0.00054698 | NA |
| C05447 | 3a,7a-Dihydroxy-5b-cholest-24-enoyl-CoA | NA | 1.99 x 10-06 | NA |
| C01259 | 3-Hydroxy-N6, N6, N6-trimethyl-L-lysine | NA | 0.0017165 | NA |
| C00671 | 3-Methyl-2-oxovaleric acid | NA | 0.002009 | NA |
| C03078 | 4-Guanidinobutanamide | 0.0012835 | NA | NA |
| C05947 | 4-Hydroxy-L-glutamic acid | NA | NA | 0.0015319 |
| C14772 | 5,6-DH x 10T | NA | NA | 0.00014131 |
| C05578 | 5,6-Dihydroxyindole | 0.00076633 | NA | NA |
| C03087 | 5-Acetamidovalerate | NA | NA | 0.0022539 |
| C05635 | 5-Hydroxyindoleacetic acid | 0.0010958 | NA | NA |
| C00643 | 5-Hydroxy-L-tryptophan | NA | NA | 0.00121 |
| C04874 | 7,8-Dihydroneopterin | 0.00068349 | NA | NA |
| C00024 | Acetyl-CoA | NA | 0.0071652 | NA |
| C06508 | Adenosyl cobinamide | NA | NA | 0.0017047 |
| ^a^C00141 | alpha-Ketoisovaleric acid | 0.0026428 | 0.0017283 | NA |
| C11695 | Anandamide | NA | 1.95 x 10-08 | NA |
| C00523 | Androsterone | NA | 9.04 x 10-05 | NA |
| C11135 | Androsterone glucuronide | 0.00067195 | NA | NA |
| C03626 | Asymmetric dimethylarginine | NA | 7.16 x 10-06 | NA |
| C08261 | Azelaic acid | NA | NA | 0.0024283 |
| ^b^C06423 | Caprylic acid | NA | 0.0038256 | 1.46 x 10-05 |
| C00386 | Carnosine | NA | NA | 0.00029098 |
| C00417 | cis-Aconitic acid | 0.0012779 | NA | NA |
| ^b^C00327 | Citrulline | NA | 0.0015296 | 0.0020241 |
| C00590 | Coniferyl alcohol | 0.00054554 | NA | NA |
| C00735 | Cortisol | NA | NA | 0.0023596 |
| C00300 | Creatine | NA | 3.84 x 10-05 | NA |
| C00063 | Cytidine triphosphate | 0.0013559 | NA | NA |
| ^a^C04555 | Dehydroepiandrosterone sulfate | 0.00032266 | 0.00818 | NA |
| C00721 | Dextrin | NA | 0.0044433 | NA |
| C01260 | Diadenosine tetraphosphate | NA | 7.80 x 10-10 | NA |
| C03242 | Dihomo-gamma-linolenic acid | NA | NA | 3.58 x 10-05 |
| C12126 | Dihydroceramide | 4.80 x 10-05 | NA | NA |
| C00906 | Dihydrothymine | NA | NA | 4.8228e-05 |
| ^a^C06196 | dIMP | 0.0011864 | 4.0899e-05 | NA |
| C00121 | D-Ribose | 1.78 x 10-05 | NA | NA |
| C00525 | D-Tryptophan | NA | 0.008309 | NA |
| C00365 | dUMP | 0.00018531 | NA | NA |
| C00503 | x 10rythritol | 0.00094339 | NA | NA |
| C02538 | x 10strone sulfate | NA | 0.0082568 | NA |
| ^a^C00439 | Formiminoglutamic acid | 2.10 x 10-05 | 0.0084442 | NA |
| C00334 | gamma-Aminobutyric acid | NA | 0.0074213 | NA |
| C06136 | Ganglioside GA1 (d18:1/12:0) | NA | 0.00033541 | NA |
| C06135 | Ganglioside GA2 (d18:1/12:0) | NA | NA | 5.54 x 10-06 |
| ^b^C06133 | Ganglioside GD3 (d18:1/12:0) | NA | 0.00025569 | 1.05 x 10-05 |
| ^b^C04911 | Ganglioside GM1 (18:1/12:0) | NA | 0.0051452 | 0.00019637 |
| C04884 | Ganglioside GM2 (d18:1/12:0) | NA | NA | 5.78 x 10-05 |
| C00325 | GDP-L-fucose | NA | 0.00024321 | NA |
| C00025 | Glutamic acid | 0.00038702 | NA | NA |
| C00064 | Glutamine | 0.0011633 | NA | NA |
| C00670 | Glycerophosphocholine | 0.00016597 | NA | NA |
| C01921 | Glycocholic acid | NA | 0.00010723 | NA |
| C01586 | Hippuric acid | 0.0013186 | NA | NA |
| ^a^C00135 | Histidine | 6.6203e-05 | 0.0015711 | NA |
| C05582 | Homovanillic acid | 0.00016022 | NA | NA |
| C00283 | Hydrogen sulfide | NA | 1.2788 10-06 | NA |
| C00519 | Hypotaurine | NA | 0.0050482 | NA |
| C00262 | Hypoxanthine | 0.00026102 | NA | NA |
| C00294 | Inosine | 0.0010484 | NA | NA |
| C00130 | Inosinic acid | NA | 0.00020894 | NA |
| ^b^C00233 | Ketoleucine | NA | 0.00090944 | 0.0015428 |
| C05396 | Lactose 6-phosphate | 0.00051276 | NA | NA |
| C00062 | L-Arginine | NA | 0.00092267 | NA |
| ^a^C00491 | L-Cystine | 0.00072667 | 3.73 x 10-05 | NA |
| C01165 | L-Glutamic gamma-semialdehyde | 0.00031648 | NA | NA |
| C02592 | Lithocholyltaurine | NA | 0.0022166 | NA |
| C00508 | L-Ribulose | 0.00014081 | NA | NA |
| C00183 | L-Valine | 0.00072784 | NA | NA |
| ^b^C00047 | Lysine | NA | 0.00020338 | 0.00072616 |
| C01835 | Maltotriose | NA | 0.0051051 | NA |
| C00073 | Methionine | 0.0012847 | NA | NA |
| ^a^C16622 | N,N'-Diacetylhydrazine | 0.001963 | 6.10 x 10-06 | NA |
| C01045 | N-Formyl-L-glutamic acid | NA | 0.0068418 | NA |
| C00712 | Oleic acid | NA | 3.02 x 10-06 | NA |
| C00346 | O-Phosphoethanolamine | NA | 0.0085406 | NA |
| C00295 | Orotic acid | NA | 0.00015121 | NA |
| C00322 | Oxoadipic acid | NA | 6.18 x 10-05 | NA |
| C00154 | Palmityl-CoA | NA | 0.008478 | NA |
| ^b^C04756 | P x 10(P-16:0/16:0) | NA | 1.16 x 10-06 | 6.26 x 10-07 |
| C00146 | Phenol | NA | 9.77 x 10-06 | NA |
| C02305 | Phosphocreatine | 0.0015346 | NA | NA |
| C05953 | Prostaglandin A2 | NA | 0.0063173 | NA |
| C06439 | Prostaglandin x 103 | 2.87 x 10-05 | NA | NA |
| C01079 | Protoporphyrinogen IX | NA | 0.0032042 | NA |
| C02737 | PS(16:0/16:0) | NA | 0.00079824 | NA |
| C15587 | Purine | 0.0026139 | NA | NA |
| C00065 | Serine | NA | 0.0023755 | NA |
| C00550 | SM(d18:1/18:0) | NA | 0.003284 | NA |
| C06124 | Sphingosine 1-phosphate | NA | NA | 8.79 x 10-07 |
| C00042 | Succinic acid | 0.0024055 | NA | NA |
| C00091 | Succinyl-CoA | NA | NA | 1.98 x 10-08 |
| ^b^C00245 | Taurine | NA | 0.001887 | 2.35 x 10-06 |
| C11134 | Testosterone glucuronide | NA | 0.001984 | NA |
| C01755 | Thiocyanate | 0.0015797 | NA | NA |
| C05963 | Thromboxane B2 | NA | NA | 2.85 x 10-05 |
| C01829 | Thyroxine | NA | 8.27 x 10-05 | NA |
| C04221 | trans-1,2-Dihydrobenzene-1,2-diol | NA | 0.00012908 | NA |
| ^a^C00086 | Urea | 0.00017514 | 3.5694e-06 | NA |
| C02355 | Uridine 2',3'-cyclic phosphate | 0.00062504 | NA | NA |
| C00029 | Uridine diphosphate glucose | 8.17 x 10-05 | NA | NA |
| C05584 | Vanillylmandelic acid | NA | NA | 6.21 x 10-06 |
| C14039 | Vinylidene chloride | NA | 0.004368 | NA |

^a^Metabolites identified in urine and plasma samples.

^b^Metabolites identified in plasma and serum samples.
